# Dual Effect of Steric Hindrance in Non‐Aqueous Amine Absorbents: Navigating the Trade‐Off Between Kinetics and Thermodynamics for Efficient CO_2_ Capture

**DOI:** 10.1002/advs.74242

**Published:** 2026-02-08

**Authors:** Xiaoyi Gao, Bin Xu, Xi Tang, Muyi Li, Cong Luo, Fan Wu, Xiaoshan Li, Liqi Zhang, Wen Chen

**Affiliations:** ^1^ State Key Laboratory of Coal Combustion School of Energy and Power Engineering Huazhong University of Science and Technology Wuhan Hubei China

**Keywords:** CO_2_ capture, non‐aqueous solvent, organic amine absorbent, quantitative structure‐activity relationship, steric hindrance effect

## Abstract

Sterically hindered amines (SHAs) offering high theoretical capacity and reduced regeneration energy, yet their molecular mechanism, especially in non‐aqueous solvents, remains unclear. This study bridges this gap by combining computational and experimental approaches. This involved a systematic investigation of diverse amine classes. We introduced a set of molecular descriptors to quantify the steric hindrance effect, correlated them with key structural features, and rigorously linked these descriptors to the absorption performance through Quantitative Structure‐Activity Relationship (QSAR) analyses, encompassing capacity, rate, and reaction thermodynamics. A systematic investigation reveals that the molecular features, including of substituent type and number, hydrogen bonding, and ring structures, affect steric hindrance. More importantly, a dual effect of steric hindrance was proposed: Steric hindrance alters the conventional zwitterionic mechanism, shifting the reaction toward an alcoholysis pathway. This substitution enhances the thermodynamic process by promoting the conversion of carbamate into alkyl carbonate, thereby raising the theoretical CO_2_ loading to 1 mol/mol. At the same time, it suppresses the kinetic process by reducing the collision efficiency of CO_2_. These fundamental understanding provide design principles for novel absorbents based on SHAs with high CO_2_ capacity and low regeneration energy requirements, paving the way for more efficient carbon capture technologies.

## Graphical abstract

This study elucidates how steric hindrance in amines creates a dual effect in CO_2_ capture: it thermodynamically favors the reaction by shifting the pathway from carbamate to alkyl carbonate formation, increasing capacity and lowering regeneration heat, while simultaneously slightly slowing the kinetics. These molecular insights provide clear design principles for developing efficient, sterically hindered amine absorbents with optimized performance.

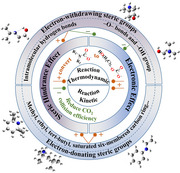

## 1 Introduction

Carbon capture, utilization, and storage (CCUS) is widely recognized as a critical strategy for mitigating carbon emissions from fossil energy sources [1]. Among CCUS approaches, amine‐based chemical absorption represents a pivotal technology for capturing CO_2_ from stationary emission sources, with widespread applications in power generation, the iron and steel industries, and other energy‐intensive sectors [2, 3].

The current industrial application predominantly utilizes second‐generation absorbents—blended amine solutions, which exhibit significant advantages over first‐generation monoethanolamine (MEA, 30 wt.% aqueous solution) in terms of improved capture efficiency and reduced regeneration energy demand [4, 5]. For example, the Petra Nova project (USA) reported a regeneration energy demand of 2.40 GJ/t CO_2_ using the KS‐1 solution [6], corresponding to an approximately 36.8% reduction relative to the MEA benchmark (∼3.8 GJ/t CO_2_). However, the high cost of CO_2_ capture continues to impede the large‐scale deployment of amine‐based chemical absorption.

To overcome the bottleneck of high energy consumption during absorbent regeneration, research efforts have increasingly focused on third‐generation absorbents [7]. Representative systems include biphasic amine [8, 9] and non‐/low‐aqueous absorbents. In non /low‐aqueous absorbents, water is partially or completely replaced by organic solvents with high boiling points and low specific heat capacities. This substitution markedly reduces solvent evaporation and achieves energy savings primarily by lowering the latent heat (*Q*_lat_) and sensible heat (*Q*_sen_) [10, 11].

**FIGURE 1:**
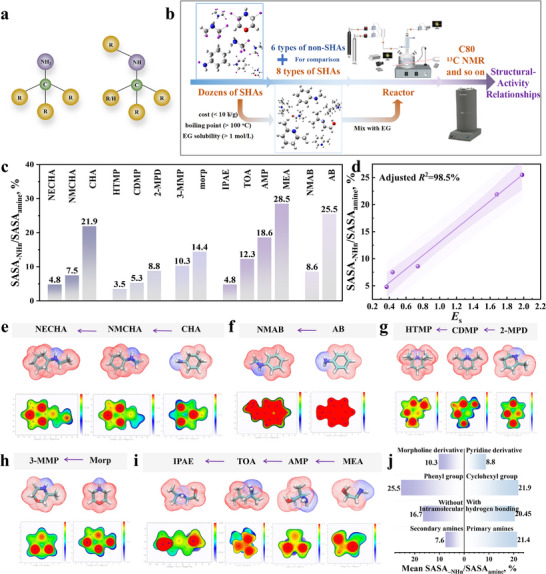
Analysis of steric hindrance effects. (a) Representative molecular configuration of SHAs; (b) Experimental workflow; (c) SASA_–NHn_/SASA_amine_ ratios of organic amines; (d) Correlation between SASA_–NHn_/SASA_amine_ and *E*_s_; Surface distance projection maps and vdW SA of (e) Cyclic amines (NECHA, NMCHA, CHA); (f) Aniline derivatives (NMAB, AB); (g) Pyridine derivatives (HTMP, CDMP, 2‐MPD); (h) Morpholine derivatives (3‐MMP, Morp); (i) Acyclic alkan(ol)amines (IPAE, TOA, AMP, MEA); (j) Comparison of mean SASA_–NHn_/SASA_amine_ ratio across different amine categories.

In contrast, the reaction heat (*Q*_rea_) is primarily governed by the amine molecular structure and reaction pathway [12]. Based on the number of substituents on the amino group, organic amines are classified as primary, secondary, or tertiary amines. Primary and secondary amines yield carbamates (${\mathrm{RNCO}}_{2}^{-}$) and protonated amines (${\mathrm{RNH}}_{2}^{+}$) via the zwitterionic mechanism, with a theoretical CO_2_ loading of 0.5 mol CO_2_/mol amine (mol/mol) [13, 14]. Tertiary amines generate bicarbonates (${\mathrm{HCO}}_{3}^{-}$) via a base‐catalyzed hydrolysis mechanism, with a higher theoretical CO_2_ loading of 1 mol/mol in aqueous solution. However, this improvement comes at the expense of substantially lower absorption rates [15]. Sterically hindered amines (SHAs) constitute a distinct subclass of primary and secondary amines, defined as “a primary amine with its amino group bound to a tertiary carbon atom, or a secondary amine with its amino group attached to a secondary or tertiary carbon atom” (Figure 1a) [16]. McCann et al. [17]. demonstrated that steric hindrance reduces carbamate stability. This destabilization facilitates carbamate hydrolysis, increasing the theoretical CO_2_ loading of SHAs to 1 mol/mol, while concurrently preserving favorable absorption rates (Figure 1a) [18].

As the most representative SHA, 2‐amino‐2‐methyl‐1‐propanol (AMP) is extensively employed as a regeneration modulating agent to lower regeneration temperature and improve efficiency, thereby reducing energy consumption. For instance, Zhou et al. [19]. demonstrated that incorporating AMP into a liquid–liquid biphasic amine absorbent consisting of diethylenetriamine (DETA), pentamethyldiethylenetriamine (PMDETA), and water increased the CO_2_ cyclic capacity from 1.85 to 4.28 mol/L. Zhu et al. [20]. reported that increasing AMP concentration from 0.5 to 1.5 mol/L in a mixed amine system composed of tetraethylenepentamine (TEPA), AMP, and water enhanced the total CO_2_ desorption capacity by a factor of 2–3. Barzagli et al. [21]. systematically compared the CO_2_ capture performance of piperazine (PZ)‐based water‐lean absorbents with and without AMP, demonstrating that the synergistic regulation of AMP not only significantly enhanced the cyclic capacity but also reduced the regeneration temperature to 80°C–90°C.

In non‐aqueous systems of SHAs, unstable can react with certain alcoholic solvents (e.g., methanol (Meth), ethanol (Eth), ethylene glycol (EG)) to form alkyl carbonates (${\mathrm{ROCO}}_{2}^{-}$), thereby achieving theoretical CO_2_ loadings of ∼1 mol/mol [22]. For example, Zheng et al. [23]. reported that the AMP–EG system achieved a CO_2_ loading greater than 0.8 mol/mol under ambient pressure. Barzagli et al. [24]. obtained a CO_2_ loading of 0.81 mol/mol in a quaternary system comprising 2‐(methylamino)ethanol (MMEA)–AMP–EG–Meth, with a regeneration efficiency as high as 95.9% at 80°C. Liu et al. [25]. improved the five‐cycle regeneration efficiency from 55.8% to 93.5% in a 1,8‐diazabicyclo[5.4.0]undec‐7‐ene (DBU)–Eth switchable ionic liquid by introducing AMP as a modulator. These findings indicate that, compared with SHA aqueous systems, non‐aqueous systems retain the advantages of high absorption capacity and superior regeneration efficiency. In addition, the high boiling points and low specific heat capacities of non‐aqueous solvents contribute to reducing the *Q*_lat_ and *Q*_sen_ of the absorbent, thereby further lowering regeneration energy consumption. For example, Lu et al. [26]. developed a blended solvent of N,N‐dimethylformamide (DMF)–EG (4:3 mass ratio), which reinforced the high absorption capacity of AMP (0.82 mol/mol) and maintained a regeneration efficiency of nearly 94% of the maximum CO_2_ adsorption capacity after seven cycles. This system achieved a regeneration energy consumption of 2.06 GJ/t CO_2_, representing a 45.8% improvement in energy efficiency relative to the benchmark MEA solution.

Furthermore, and have been shown to exhibit weaker interactions with solvent molecules than ${\mathrm{RNCO}}_{2}^{-}$ [19]. Consequently, SHAs have also been employed as modulators to reduce the viscosity of saturated absorbents. For instance, Geng et al. [27]. introduced AMP into a self‐constructed biphasic absorbent, reducing the viscosity of the CO_2_‐rich liquid phase from 527.0 to 92.3 mPa·s. Meanwhile, SHAs exhibit strong thermal stability, which mitigates absorbent corrosiveness and degradation [21].

As illustrated by the examples discussed earlier, current research on SHAs has been largely centered on AMP, leaving the influence of molecular substructures on steric hindrance effects, as well as the consequent impact on CO_2_ capture performance, insufficiently understood.

To address this gap, we developed a series of novel SHAs with diverse molecular structures, and also introduced several non‐SHAs for comparative analysis. By integrating quantum chemical calculations with batch absorption–desorption experiments, we systematically investigated the quantitative structure–activity relationships (QSAR) between molecular structure, steric hindrance, and CO_2_ capture performance. The overall experimental flowchart is illustrated in Figure 1b. Within this framework, first‐principles insights into steric and electronic effects are complemented by pathway analysis, together revealing the mechanistic origins of reactivity and thermophysical behavior. Collectively, this work provides a deeper mechanistic understanding of SHAs and guidance for the rational design of next‐generation CO_2_ absorbents.

## 2 Results and Discussion

### 2.1 Steric Hindrance Effect of Organic Amines

The steric hindrance effect primarily arises originates from spatial obstruction imposed by adjacent atoms or functional groups within a molecule. The steric effects of amine molecules were visualized using surface distance projection maps and van der Waals surface area (vdW SA) analyses. In vdW SA, blue and red distinguish between the amino group and the molecular backbone. In surface distance projection maps, a blue‐to‐red gradient represents a continuous decrease in distance to the projection surface. Although the Taft steric parameter (*E*_s_) is widely applied to quantify steric effects, it was not adopted here because it cannot adequately characterize complex substituents such as morpholine and pyridine derivatives. Experimentally derived *E*_s_ values were available only for the organic amines N‐Ethylcyclohexylamine (NECHA), N‐Methylcyclohexylamine (NMCHA), cyclohexylamine (CHA), N‐Methylaniline (NMAB), and Aniline (AB), with the calculated results shown in Figure S1 and corresponding substituent values listed in Table S1. Consequently, solvent‐accessible surface area (SASA) was employed to quantify steric hindrance, where a smaller SASA of the amino group (–NH_n_) indicates stronger steric effects. The relative contribution of the –NH_n_ SASA to the total amine SASA (SASA_–NHn_/SASA_amine_) is presented in Figure 1c. Validation revealed an excellent correlation between SASA and *E*_s_ (adjusted *R* ^2^ = 0.985, Figure 1d), confirming the robustness of this methodology.

In this study, organic amines were categorized into acyclic alkan(ol)amines (2‐(Isopropylamino)ethanol (IPAE), *tert*‐Octylamine (TOA), AMP, MEA), cyclic amines (NECHA, NMCHA, CHA), morpholine derivatives (3‐Methylmorpholine (3‐MMP), Morpholine (Morp)), pyridine derivatives (2,2,6,6‐Tetramethylpiperidine (HTMP), *cis*‐2,6‐Dimethylpiperidine (CDMP), 2‐Methylpiperidine (2‐MPD)), and aniline derivatives (NMAB, AB) based on molecular type. Although morpholine, pyridine, and aniline derivatives are subclasses of cyclic amines, they were treated as independent categories to facilitate comparative analysis of how distinct ring architectures affect steric hindrance and CO_2_ capture performance.

#### 2.1.1 Linear Alkyl Substituents

As shown in Figure 1e, steric hindrance increases progressively as substituents on the amino nitrogen atom change from ─H to a methyl group (─CH_3_) and then to an ethyl group (─CH_2_CH_3_), indicating that steric effects intensify with increasing length of linear alkyl substituents. This increase is especially pronounced in the transition from ─H to ─CH_3_, where CHA exhibited a SASA_–NHn_/SASA_amine_ ratio of 21.9%, which was 2.9 times higher than that of NMCHA (7.5%). A similar trend was observed for NMAB (8.6%) and AB (25.5%) (Figure 1f), confirming that secondary amines exhibit substantially stronger steric hindrance than primary amines. The primary amines examined in this study (CHA, AB, MEA, AMP, and TOA) exhibited the highest SASA_–NHn_/SASA_amine_ ratios, averaging 21.4%, whereas secondary amines showed a mean ratio of only 7.6%. This observation is consistent with findings by Deng et al. [28], who reported that the introduction of a second alkyl group markedly restricts access to the reactive nitrogen site.

Characterization of steric hindrance in 2‐MPD (8.8%), CDMP (5.3%), and HTMP (3.5%) (Figure 1g) shows a progressive increase as the number of methyl substituents at the α‐carbon rises from one to four, thereby establishing a positive correlation between substituent number and steric magnitude. Figure 1h,i further confirm this positive correlation: 3‐MMP (one methyl substituent, 10.3%) exhibits stronger steric hindrance than Morp (no substituent, 14.4%), while AMP (two methyl substituents, 18.6%) shows higher steric hindrance than MEA (no substituent, 28.5%).

#### 2.1.2 Intramolecular Hydrogen Bonding

The acyclic alkanolamines MEA, AMP, and IPAE contain hydroxyl groups (─OH), where intramolecular hydrogen bonds are formed between ─OH and ─NH_n_ in MEA and IPAE (Figure S2). To examine the steric contributions of intramolecular hydrogen bonding, dehydroxylated derivatives—ethylamine (EA) and N‐isopropylethylamine (IPA)—were generated for comparative analysis. The SASA_─NHn_/SASA_amine_ ratios for EA and IPA were 33.9% and 7.0%, respectively, corresponding to 1.19‐fold and 1.46‐fold increases compared with their parent molecules MEA (28.5%) and IPAE (4.8%). A comparison of vdW SA before and after modification is presented in Figure S3. These results demonstrate that intramolecular hydrogen bonds involving –NH_n_ significantly enhance the steric hindrance of the molecule.

Notably, although MEA is not conventionally classified as a SHA, it nonetheless exhibits measurable steric hindrance effects. This indicates that, unlike SHAs where steric hindrance imposes strict configurational constraints, varying degrees of steric hindrance are broadly present across amine molecules.

#### 2.1.3 Six‐Membered Carbon Ring Substituents/Heterocyclic Compounds

Both CHA and AB contain six‐membered carbon ring substituents on their ─NH_2_ group: a cyclohexyl group (─C_6_H_11_) in CHA and a phenyl group (─C_6_H_5_) in AB. The SASA_–NHn_/SASA_amine_ ratios of 21.9% (CHA) and 25.5% (AB) indicate that the ─C_6_H_11_ group imposes substantially stronger steric hindrance than the ─C_6_H_5_ group. This difference arises because the planar structure of the ─C_6_H_5_ group reduces steric repulsion, whereas the chair conformation of the ─C_6_H_11_ group, along with its axial hydrogen atoms, generates pronounced steric constraints.

Both 2‐MPD (a pyridine derivative) and 3‐MMP (a morpholine derivative) are heterocyclic compounds containing an endocyclic ─NH group. Their SASA_–NHn_/SASA_amine_ ratios of 8.8% (2‐MPD) and 10.3% (3‐MMP) indicate that the piperidine derivative exhibits slightly stronger steric hindrance. Structural analysis shows that replacing the C4 atom in 2‐MPD with an O atom to generate a morpholine ring increases the dihedral angle (atoms 1–4) from 56.1° to 57.4°, and the N─C2 (α carbon)─C5 (α‐substituent) bond angle from 109.9° to 110.8°. These changes reduce the spatial density of surrounding atoms, thereby lowering the steric effect (Figure S4). A comparative summary of the different amine types is shown in Figure 1j.

### 2.2 QSAR between Steric Hindrance Effects and Absorption Performance

AB and NMAB were excluded due to their negligible CO_2_ capture capacity, which arises from p‐π conjugation between the nitrogen lone pair and the aromatic π‐system. This electronic delocalization markedly reduces the nucleophilicity of ─NH_n_. A similar electronic effect is observed in morpholine derivatives (Morp and 3‐MMP), where the higher electronegativity of oxygen relative to nitrogen withdraws electron density, resulting in reduced loadings (*L*_Morp_ = 0.50 mol/mol, *L*_3‐MMP_ = 0.37 mol/mol).

#### 2.2.1 QSAR between Steric Hindrance Effects and Absorption Rates

**FIGURE 2:**
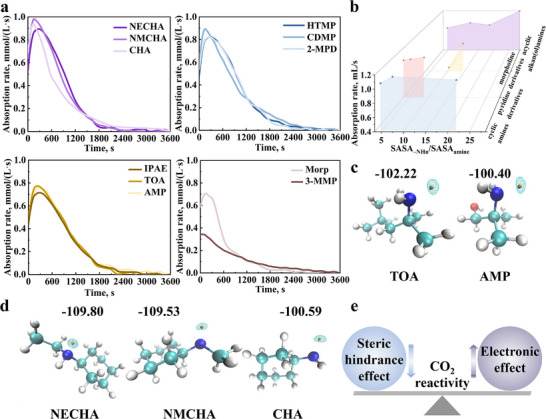
QSAR analysis of absorption rate and viscosity. (a) CO_2_ absorption rate of organic amines; (b) Relationship between absorption rate and SASA_–NHn_/SASA_amine_ across different amine classes; (c) *V*_ESP, min_ of TOA and AMP; (d) *V*_ESP, min_ of cyclic amines (NECHA, NMCHA, CHA); (e) Impact on CO_2_ reactivity.

The CO_2_ absorption rates of the investigated amines are shown in Figure 2a, with the quantitative relationship between steric hindrance and reaction rate presented in Figure 2b. In general, absorption rates increase with higher SASA_–NHn_/SASA_amine_ ratios, indicating that stronger steric hindrance reduces collision efficiency with CO_2_, and thereby suppressing uptake rates. Notable exceptions are CHA and AMP, which deviate from this trend. To clarify these anomalies, steric and electronic effects [29] were jointly analyzed.

As shown in Figure 2c, both TOA and AMP exhibit ESP minima localized at the lone pair electrons of the N atoms (the reactive sites), with values of −102.22 and −100.40 kcal/mol, respectively. This difference arises from substituent effects at the β‐carbon: the tert‐butyl group (─C(CH_3_)_3_) in TOA acts as an electron‐donating group, whereas the ─OH group in AMP is electron‐withdrawing, thereby lowering the nucleophilicity of AMP and reducing its reaction rate. Similarly, Figure 2d shows that compared with CHA, NECHA and NMCHA incorporate ethyl (─CH_2_CH_3_) and methyl (─CH_3_) substituents, respectively. These electron‐donating groups decrease *V*_ESP, min_ from −100.59 kcal/mol in CHA to −109.80 and −109.53 kcal/mol in NECHA and NMCHA. Consequently, although CHA has a higher SASA_–NHn_/SASA_amine_ ratio (indicating weaker steric hindrance), its absorption rate is lower than NMCHA due to reduced nucleophilicity in the absence of electron‐donating substituents.

Collectively, these findings demonstrate that the CO_2_ reactivity of organic amines is co‐regulated by both spatial and electronic factors: steric hindrance impedes molecular collisions, reducing reaction rates, whereas electron‐donating substituents enhance nucleophilicity, promoting reactivity (Figure 2e).

#### 2.2.2 QSAR Analysis of Steric Hindrance Effects and Absorption Loads

**FIGURE 3:**
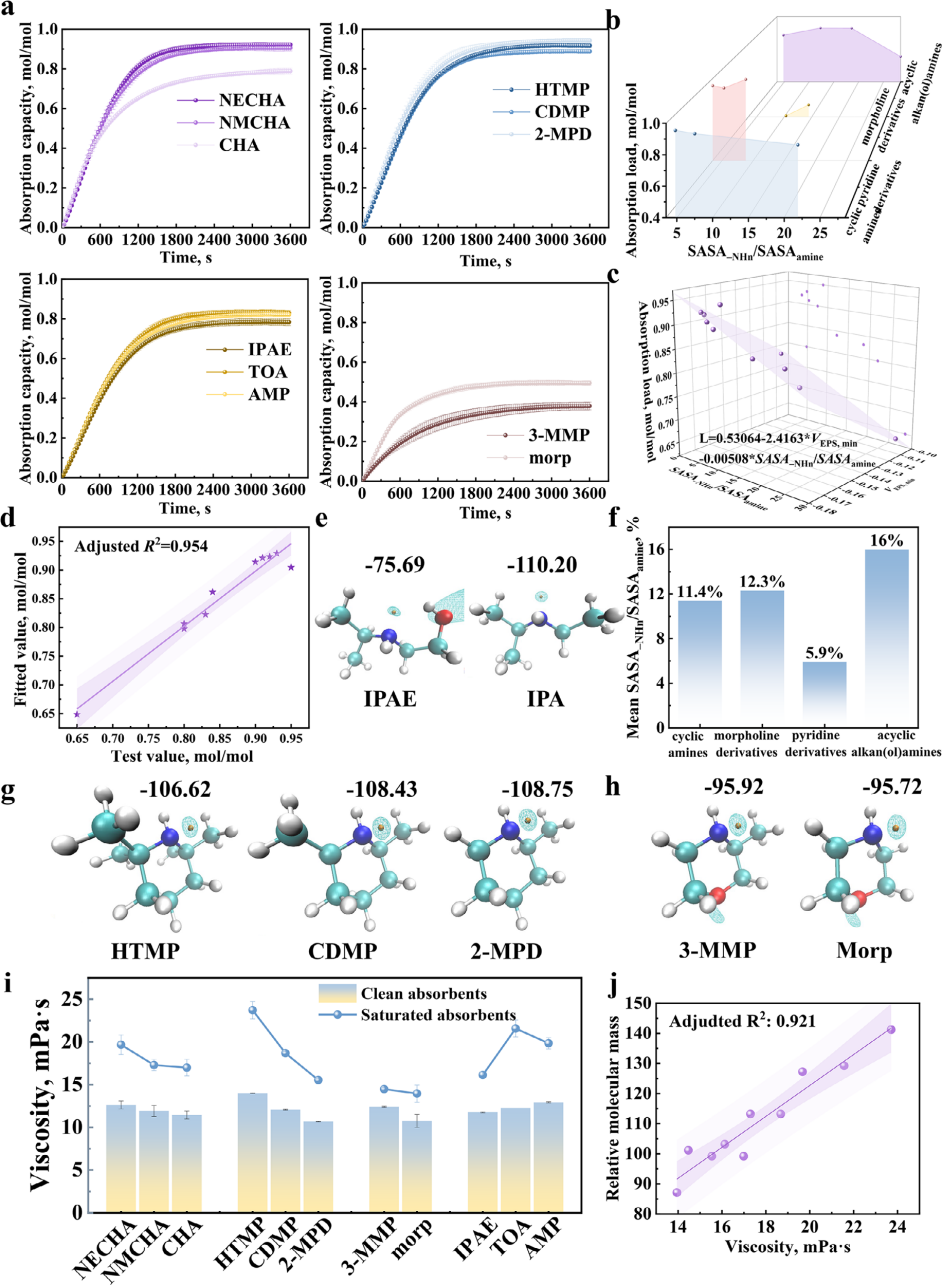
QSAR analysis of CO_2_ absorption capacity. (a) CO_2_ loading of different amines; (b) Variation of CO_2_ loading with SASA_–NHn_/SASA_amine_ across amine classes; (c) 3D regression surface of fitted model; (d) Comparison between experimental and fitted loading values; (e) *V*_ESP, min_ of IPAE and IPA; (f) Mean SASA_–NHn_/SASA_amine_ ratios for different amines types; (g) *V*_ESP, min_ of pyridine derivatives (HTMP, CDMP, 2‐MPD); (h) *V*_ESP, min_ of morpholine derivatives (3‐MMP and Morp). (i) Comparison of viscosities among different amine classes; (j) Correlation between viscosity and molecular mass.

The CO_2_ absorption loadings of the organic amines are presented in Figure 3a. The non‐SHAs CHA and MEA exhibited loadings of 0.80 and 0.65 mol/mol, respectively, both exceeding the theoretical maximum of 0.5 mol/mol. This aligns with the conclusion in Section 2.1 that steric effects imposed by the saturated six‐membered rings in CHA and by intramolecular H‐bonding in MEA remain significant, even though neither conforms to the classical definition of SHAs. In contrast, cyclic and piperidine‐based SHAs achieved high loadings (L≥0.9 mol/mol).

The relationship between CO_2_ loading and steric hindrance is illustrated in Figure 3b, revealing pronounced heterogeneity among amine classes. To elucidate the influence of steric hindrance on absorption performance, the *V*_ESP, min_ was calculated to represent the electronic effects for a synergistic analysis. Interestingly, distinct patterns emerged when CO_2_ loading with *V*_ESP, min_ were quantitatively integrated (excluding morpholine derivatives). The fitting results are shown in Figure 3c,d, and the fitted equation is as follows:

$$L=0.53064-0.00508\times SASA_{-\mathrm{NHn}}/SASA_{\mathrm{amine}}-2.4163\times V_{\mathrm{ESP},\;\min}$$

According to the fitting results, CO_2_ loading shows a positive correlation with steric hindrance (represented by a lower *SASA*_–NHn_/*SASA*_amine_ ratio) and electronic effects (represented by a more negative *V*_ESP, min_). Electronic effects enhance N‐nucleophilicity during CO_2_ capture, while steric hindrance promotes amine utilization by destabilizing carbamate. Consequently, in cyclic amines (CHA → NECHA → NMCHA), the concurrent increase in steric hindrance (*SASA*_–NHn_/*SASA*_amine_: 21.9% → 4.8%) and electronic effects (*V*_ESP, min_: −100.59 → −109.53 kcal/mol) raises CO_2_ loading from 0.80 to 0.95 mol/mol. A similar trend is observed for acyclic alkan(ol)amines (MEA, AMP, and TOA), with IPAE representing an exception due to ─OH─mediated intramolecular H─bonding that markedly attenuates electronic effects (Figure 3e). Thus, CO_2_ absorption capacity is governed by the synergistic interplay of steric hindrance and electronic factors [30]. When steric substituents are electron‐withdrawing, the diminished electronic contribution outweighs steric benefits, leading to reduced CO_2_ loading.

A similar phenomenon occurs in piperidine derivatives, which exhibit the strongest steric hindrance (mean *SASA*_–NHn_/*SASA*_amine_ ratio = 5.9%, Figure 3f). However, excessive sterics compression induces hyperconjugative delocalization of the nitrogen lone pair. As a result, despite an increasing number of electron‐donating ─CH_3_ groups, *V*_ESP, min_ decreases (Figure 3g), leading to a paradoxical reduction in CO_2_ loading under stronger steric constraints.

Notably, in morpholine derivatives, the stronger steric and electronic effects of 3‐MMP relative to Morp (*SASA*_–NHn_/*SASA*_amine_: 10.3% → 14.4%) reduces the maximum absorption rate from 0.81 to 0.42 mmol/(s·L). Combined with the electron‐withdrawing effect of ether bond (─O─) (Figure 2h), this kinetic penalty outweighs thermodynamic benefits, reducing loading from 0.50 to 0.37 mol/mol. Overall, while steric hindrance generally enhances CO_2_ loading, excessively strong steric effects combined with weak electronic contributions can suppress absorption performance.

#### 2.2.3 QSAR Analysis of Steric Hindrance Effects and Viscosity

Viscosity variations before and after CO_2_ absorption are shown in Figure 3i. Saturated solutions exhibit markedly higher viscosities than unloaded ones. For cyclic amines, piperidine derivatives, and morpholine derivatives, viscosity before and after CO_2_ uptake correlates positively with steric hindrance. This trend is primarily attributable to the increased molecular mass introduced by bulky steric substituents. Molecular mass‐viscosity regression yields an adjusted *R* ^2^ of 0.921 (Figure 2j; Figure S5, with AMP excluded).

Among alkanolamines, AMP exhibits an abnormally high viscosity due to its ─OH group, which serves as a hydrogen‐bond donor without forming intramolecular H─bonds. This configuration strengthens intermolecular interactions and thus elevates viscosity. By contrast, IPAE, as a secondary amine, forms intramolecular hydrogen bonds that generate steric effects comparable to bulky substituents while maintaining a relatively low molecular weight, ultimately leading to reduced viscosity.

In summary, within a given amine class, enhanced steric hindrance is generally accompanied by higher molecular mass from bulky substituents, which in turn increases solution viscosity. The influence of ─OH group on viscosity is dual: it can increase viscosity via intermolecular hydrogen bonding when intramolecular hydrogen bonds are not formed, whereas the formation of intramolecular hydrogen bonds helps reduce the viscosity of the absorbent.

### 2.3 Reaction Mechanism of SHAs

#### 2.3.1 Analysis of Product Molecular Type

**FIGURE 4:**
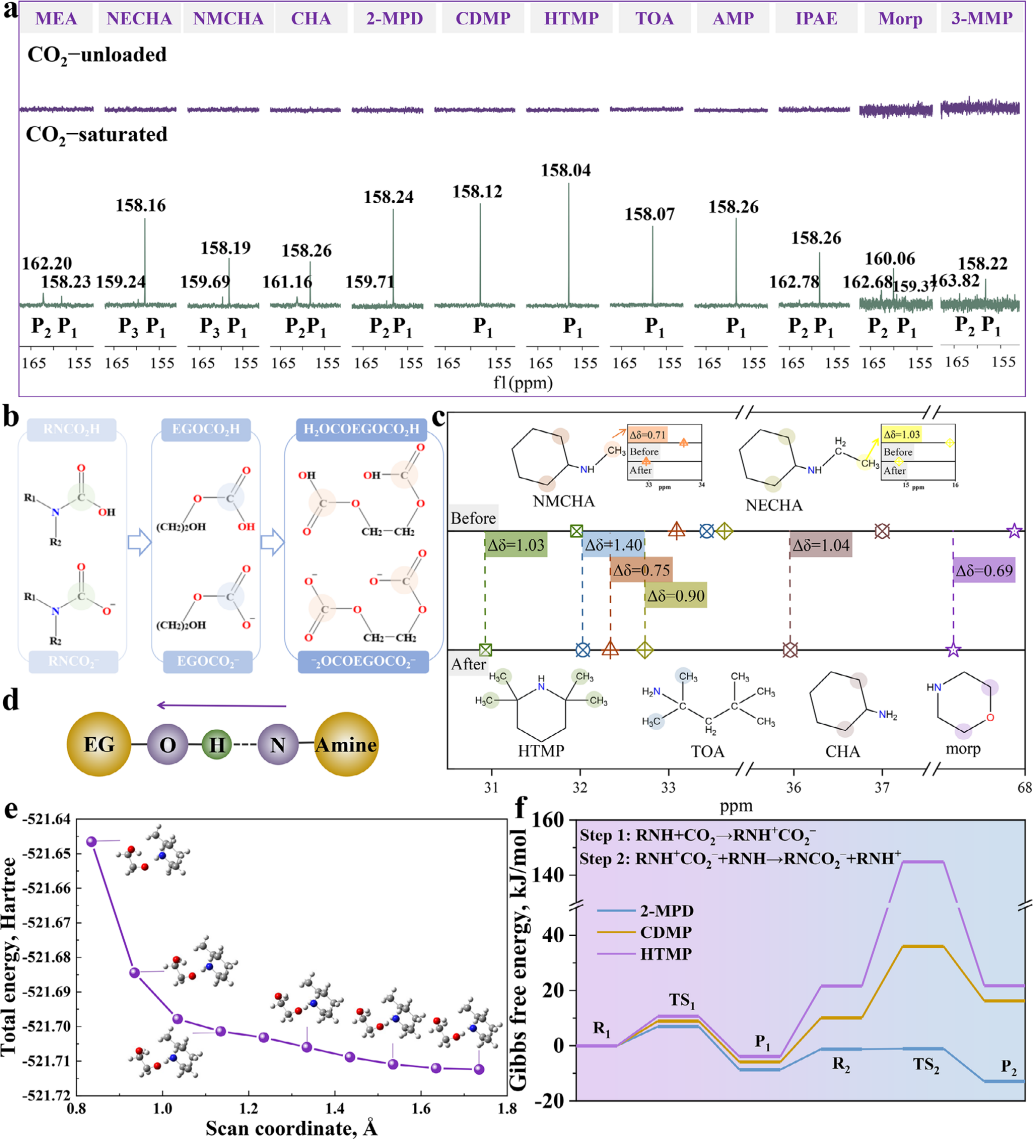
Analysis of reaction mechanism. (a) ^13^C NMR spectra in the range 155–165 ppm; (b) Molecular types of the absorption products; (c) High‐field shift of β‐carbon atoms linked to the nitrogen atom; (d) Electronic migration path; (e) Flexible scan from 2‐MPD with EG to 2‐MPDH^+^ with EGO^−^; (f) Gibbs free energy of reactants, products, and transition states based on the zwitterion mechanism.

The ^1^ ^3^C NMR spectra of the investigated amines in the range of 155–165 ppm reveal the molecular types of CO_2_ absorption products (Figure 4a,b). For MEA, IPAE, Morp, 3‐MMP, and CHA (mean loading: 0.62 mol/mol), characteristic peaks appear simultaneously at 161.16–163.82 and 158.22–159.37 ppm, indicating the coexistence of and EG‐alkyl carbonates (${\mathrm{EGOCO}}_{2}^{-}$) species. In contrast, CDMP, HTMP, TOA, and AMP (mean loading: 0.88 mol/mol) exhibit single peaks at 158.04–158.26 ppm, confirming that their main products are ${\mathrm{EGOCO}}_{2}^{-}$. Notably, NECHA, NMCHA, and 2‐MPD, with high loadings (mean loading: 0.93 mol/mol) display dual peaks at 158.22–159.37 and 159.24–159.71 ppm, signifying the concurrent presence of and EG‐dialkyl carbonate (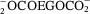).

An intriguing phenomenon was observed in the non‐ester carbon region (Figures S6–S17): when organic amines were mixed with EG, the β‐carbon atoms linked to the nitrogen atom exhibited a significant high‐field shift (Figure 4c). This shift may arise from two possible mechanisms: (1) the transfer of a H atom from the ─OH group of EG to the N atom of the amine, producing a and deprotonated EG (EGO^−^); or (2) the formation of a H─bond between the ─OH group of EG and the N atom of the amine (Figure 4d). Both mechanisms affect the NMR chemical shift of the β‐carbon by decreasing the electron density around the N atom. To elucidate the underlying process, a flexible potential energy scan was performed for 2‐MPD, simulating the gradual approach of the H atom from the ─OH group of EG toward the N atom of the amine. The results (Figure 4e) show that as the H atom approached and transferred to nitrogen, the Gibbs free energy of the system increased from −521.71 to −521.65 Hartree, indicating that proton transfer is thermodynamically unfavorable. Furthermore, geometry optimization of all with configurations converged to stable structures in which the amine and EG were connected via hydrogen bonding (Figure S18). Therefore, the high‐field shift of the β‐carbon in the NMR spectrum can be attributed to the formation of intermolecular hydrogen bonds, ruling out the possibility of protonated amine formation and the direct reaction of with CO_2_.

In summary, intermolecular hydrogen bonds form when organic amines are mixed with EG. During CO_2_ absorption, organic amines initially react with CO_2_ to form ${\mathrm{RNCO}}_{2}^{-}$ [22, 31]. As the loading increases, these intermediates are subsequently converted into, and ultimately transformed into 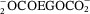.

#### 2.3.2 QSAR Analysis of Steric Hindrance Effects and Gibbs Free Energy

To further investigate the influence of steric hindrance effects on the absorption mechanism, the Gibbs free energies of reactants, products, and transition states during the CO_2_ absorption process were analyzed according to the zwitterionic mechanism, using 2‐MPD, CDMP, and HTMP as representative examples (Figure 4f). The results indicate that the energy barrier for zwitterion deprotonation is significantly higher than that for its formation, confirming that deprotonation is the rate‐determining step. With increasing steric hindrance, the energy barriers for both Step 1 and Step 2 rise progressively (Figure S19), leading to a corresponding decrease in the solvent's absorption rate. Moreover, the Gibbs free energy change (ΔG) for the overall reaction increases with the enhanced steric hindrance (Figure S20), indicating that the steric effects destabilize the carbamate product and thereby facilitates its further conversion. Notably, the ΔG of Step 2 first increases and then decreases with increasing steric hindrance, a trend consistent with the observed variation in CO_2_ loading. Collectively, these findings demonstrate that the steric hindrance modulates the CO_2_ absorption process by inhibiting reaction kinetics while promoting reaction thermodynamics.

### 2.4 Reaction Heat of SHAs

**FIGURE 5:**
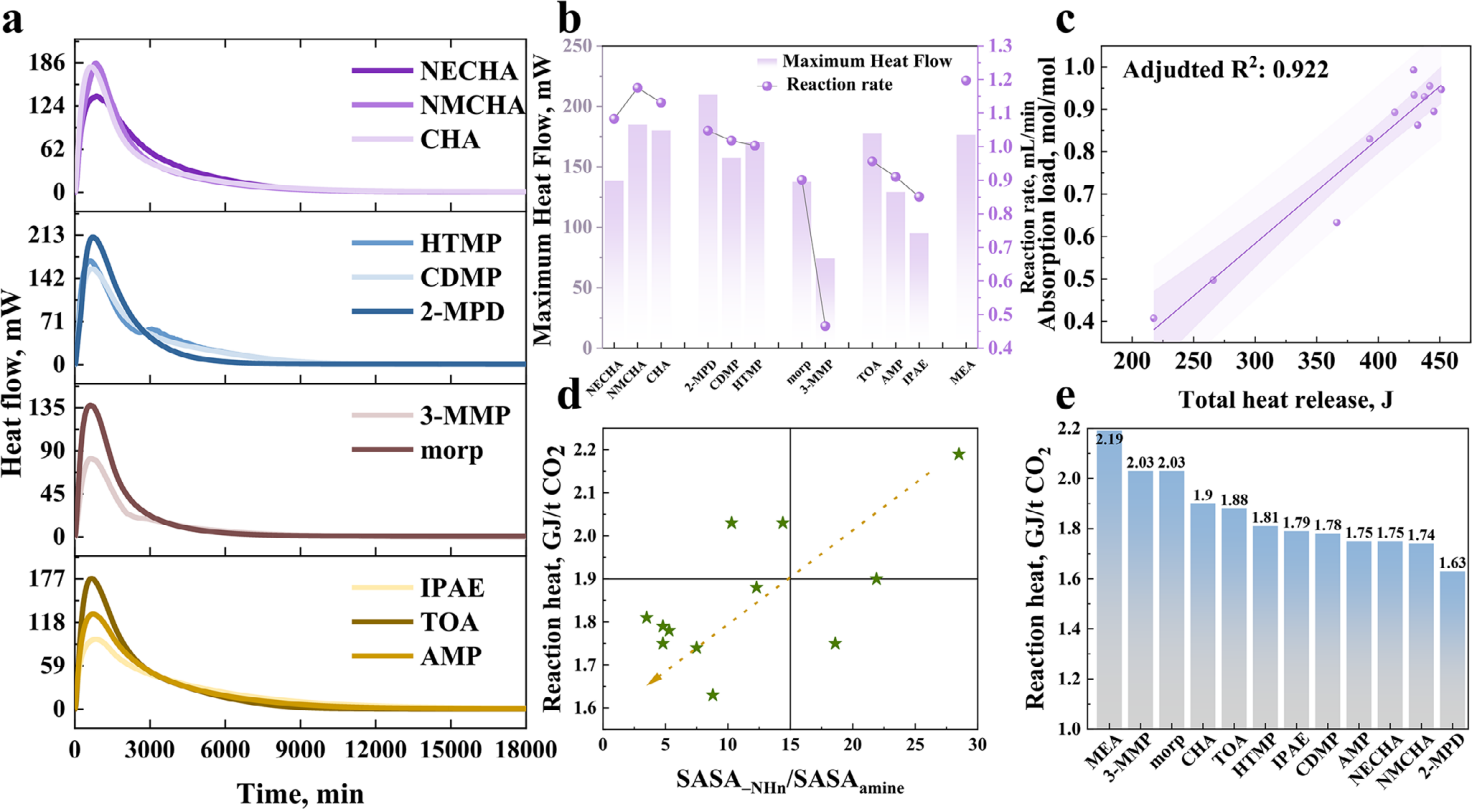
Analysis of reaction heat of absorbents. (a) Heat flow as a function of reaction time; (b) Comparison between maximum heat flow and maximum absorption rate; (c) Relationship between total heat release and absorption loading; (d) Reaction heat vs. steric hindrance; (e) Reaction heat presented in descending order.

The heat flow profiles of different organic amine absorbents as a function of reaction time are shown in Figure 5a. Upon initiation of the reaction, heat flow rises rapidly to a peak value corresponding to the fast reaction stage. As the reaction approaches completion, the heat flow gradually declines and eventually returns to baseline. Specific parameters, including maximum heat flow and total heat release, are summarized in Figures S21 and S22. The maximum heat flow follows a trend consistent with the maximum absorption rate (Figure 5b), which can be attributed to the more concentrated heat release within a shorter reaction period at higher rates. Furthermore, total heat release correlates linearly with CO_2_ loading across the different absorbents (adjusted *R* ^2^ = 0.922, Figure 5c).

As shown in Figure 5d, reaction heat generally decreases with increasing steric hindrance. The reaction heat values for each amine are presented in descending order in Figure 5e. Based on their product types, the amines can be categorized into three tiers: The first tier includes the non‐SHAs MEA and CHA, along with the morpholine derivatives Morp and 3‐MMP. These amines exhibit the four highest reaction heats (*H*_rea_>1.9 GJ/t CO_2_), and their products consist of a mixture of and ${\mathrm{EGOCO}}_{2}^{-}$. The second tier, ranging from AMP to TOA, shows a gradual increase in reaction heat from 1.75 to 1.88 GJ/t CO_2_. With the exception of IPAE, whose deviation is attributed to intramolecular H─bond [12], the reaction products in this group are ${\mathrm{EGOCO}}_{2}^{-}$. The third tier comprises the three amines with the lowest reaction heats: NECHA (1.75 GJ/t CO_2_), NMCHA (1.74 GJ/t CO_2_), and 2‐MPD (1.63 GJ/t CO_2_). Their reaction products correspond to a mixture of and 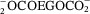. These results clearly demonstrate that modulation of the reaction pathway through steric hindrance induces a progressive transformation of products from toward and ultimately to, thereby significantly reducing the reaction heat of the absorbents.

## 3 Conclusion

The influence of substituents on steric hindrance effects was systematically investigated in this study. Unlike the rigid configurational constraints imposed by SHA, varying degrees of steric hindrance are commonly observed among general amine molecules. In particular, steric hindrance is more pronounced in secondary amines than in primary ones. Specifically, steric hindrance intensifies with both the length of linear alkyl substituents and the number of substituent groups. Intramolecular hydrogen bonding involving the ─NH_n_ group can also significantly enhance steric hindrance. Additionally, saturated six‐membered rings exhibit stronger steric hindrance than phenyl rings, while among heterocyclic compounds, piperidine derivatives display slightly greater steric hindrance than morpholine derivatives.

QSAR analyses were further conducted on absorption capacity, absorption rate, viscosity, reaction heat, and reaction mechanism. The results show that absorption loading increases with enhanced steric hindrance; however, excessively strong steric effect combined with weak electronic effects from electron‐withdrawing groups such as ─O─ bonds and intramolecular hydrogen bonds, can suppress overall absorption performance. Regarding the absorption rate, increased steric hindrance reduces the collision efficiency between the amine active site and CO_2_, thereby lowering the uptake rate. In terms of viscosity, high steric hindrance typically correlates with increased molecular mass due to bulky substituents and higher CO_2_ loading, both of which contribute to elevated viscosity. Overall, steric hindrance exerts a dual influence on CO_2_ absorption performance: it promotes thermodynamic favorability while inhibiting reaction kinetics. Mechanistically, steric effects destabilize the carbamate product, facilitating its subsequent conversion into alkyl carbonate and, eventually, di‐alkyl carbonate. This transformation coupled with the enhanced electron‐withdrawing effects of substituents drives the improvement in reaction thermodynamics. Ultimately, the strategy of modulating reaction pathways through steric hindrance to induce product conversion has been demonstrated to significantly reduce the reaction heat of the absorbents.

## 4 Experimental Section

### 4.1 Chemicals

Eight SHAs were selected from dozens of candidates based on the cost (< 10 ¥/g), boiling point (> 100°C), and solubility in ethylene glycol (≥ 1 mol/L). The candidate set spanned seven categories: acyclic alkan(ol)amines, cycloamines, anilines, piperidines, morpholines, and piperazines. The eight selected SHAs were: NECHA, NMCHA, HTMP, CDMP, 2‐MPD, IPAE, AMP, and TOA. For comparison, the non‐SHA compounds CHA and MEA were also included. Specific subclasses of cyclic amines–morpholine derivatives (3‐MMP, Morp) and aniline derivatives (NMAB, AB) were employed to probe the steric hindrance effects of ring structures. EG was used as the solvent to facilitate the conversion of to, as its high product solubility prevents precipitation [32, 33]. All organic reagents used in this study were purchased from Shanghai Aladdin Biochemical Technology Co., Ltd. and used without further purification. Detailed information on the employed chemicals is provided in Table S2, while excluded SHAs are listed in Tables S3–S5.

### 4.2 CO_2_ Absorption

Solutions of SHAs in EG were prepared uniformly at a concentration of 1 mol/L and introduced into the reactor. Pure CO_2_ gas was continuously supplied to the reactor at a constant flow rate of 80 mL/min until the outlet stream reached steady state. The reaction temperature was maintained at 313.15 K using a thermostatic magnetic stirrer (DLAB MS7‐H550‐Pro, China). The outlet flow rate was monitored and recorded using a digital mass flow controller (Sevenstar CS200, China). A Chittick apparatus was employed to further validate the CO_2_ loading measured by the mass flow controller. Schematic diagrams of the CO_2_ absorption setup and Chittick apparatus are provided in Figures S23 and S24. Viscosity was measured using a rotational viscometer (Brookfield, LVDV2T, USA) with an accuracy of ±1.0%. The *Q*_rea_ of CO_2_ absorption was determined using a microcalorimeter (C80, SETARAM, France). All experiments were conducted in triplicate, and results are reported as mean values.

### 4.3 ^13^C NMR Analysis

^13^C NMR spectroscopy (Ascend 600 mHz, Bruker, Switzerland) was employed to qualitative identify the product molecular types formed during CO_2_ capture by the absorbents. Dimethylsulfoxide‐d6 (DMSO‐d_6_) was used as the solvent for all samples requiring deuterium locking. For Morp‐containing solutions, methanol‐d_4_ (CD_3_OD) was used instead of DMSO‐d_6_. This substitution was necessary due to the limited solubility of Morp in DMSO‐d_6_.

### 4.4 Quantum Chemical Calculation

All molecular conformations in this study were identified as the lowest‐energy structures (Table S1) using Molclus and GROMACS [34, 35]. Detailed procedures and parameters are provided in Section S1. Molecular structures were further optimized, and transition states were located at the B3LYP/6‐311+G(d, p) level of theory using the Gaussian 16 [36]. Solvent effects were simulated using the universal implicit solvent model (SMD), as it can account for both the polar and non‐polar contributions. The model parameters for EG are as follows: eps = 37.0; epsinf = 2.0420; HBondAcidity = 0.90; HBondBasicity = 0.52; SurfaceTensionAtInterface = 66.91; CarbonAromaticity = 0; ElectronegativeHalogenicity = 0. To assess steric hindrance effects of the organic amines, Multiwfn was used to generate surface distance projection maps and to calculate the vdW SA and global minima of electrostatic potential (*V*_ESP, min_) [37]. VMD 1.9.3 was applied to compute the SASA and to visualize vdW SA and *V*_ESP, min_ [38]. Activation free energy (E_a_) and Gibbs free energy change (ΔG) were derived according to Equations (S1) and (S2).

## Funding

This work was financially supported by the Special Funds for Guiding Local Scientific and Technological Development by the Central Government of China (Hubei, 2024CSA088) for funding this project.

## Conflicts of Interest

The authors declare no conflicts of interest.

## Supporting information




**Supporting File**: advs74242‐sup‐0001‐SuppMat.docx.

## Data Availability

The authors declare that the main data supporting the findings of this study are available within the article and its supporting information files. Extra data are available from the corresponding authors upon reasonable request.
