# Supplementary material for: Dual Effect of Steric Hindrance in Non‐Aqueous Amine Absorbents: Navigating the Trade‐Off Between Kinetics and Thermodynamics for Efficient CO2 Capture
Source: Adv Sci (Weinh). 2026 Feb 8;13(21):e74242. doi: 10.1002/advs.74242 (PMC13073257; doi:10.1002/advs.74242)
Supplement: Supplementary file 1 — Supporting File: advs74242‐sup‐0001‐SuppMat.docx. [file ADVS-13-e74242-s001.docx]

Supporting Information for:

**Dual Effect of Steric Hindrance in Non-Aqueous Amine Absorbents: Navigating the Trade-off between Kinetics and Thermodynamics for Efficient CO_2_ Capture**

Xiaoyi Gao, Bin Xu, Xi Tang, Muyi Li, Cong Luo, Fan Wu, Xiaoshan Li*, Liqi Zhang, Wen Chen

(State Key Laboratory of Coal Combustion, School of Energy and Power Engineering, Huazhong University of Science and Technology, Wuhan, Hubei 430074, China)

Corresponding Author: [lxs0721@hust.edu.cn](mailto:lxs0721@hust.edu.cn).

**Number of pages: 34**

**Number of figures: 24**

**Number of tables: 5**

**Number of equations: 2**

**Text Materials and methods**

**S1 Conformation search**

The molecular structure parameters and wave function analysis parameters involved in this study were all calculated based on the lowest energy structure of the molecules. The process of obtaining the lowest energy structure of molecules is as follows: 1. Conformations of cyclic and chain-structured molecules were generated using the annealing function of the GROMACS program and the gentor program, respectively. 2. The Gaussian 16 package was called by the molclus program to conduct conformational searches at the B3LYP-D3(BJ)/6-31G(d) level. 3. The isomers program was used to analyze the percentage of each conformation at a temperature of 298.15 K based on the Boltzmann distribution and to identify the most stable conformation of each molecule for subsequent optimization of the molecular structure.

**S2 Free energy calculation methods**

The activation free energy (Ea, kJ/mol) and Gibbs free energy change (ΔG, kJ/mol) can be calculated using Eqs. (S1)-(S2).

$\text{E}_{\text{a}}=2625.5\times(E_{\text{transition state}}-E_{reac\tan t})$ (S1)

$\text{ΔG}=2625.5\times(E_{product}-E_{reac\tan t})$ (S2)

Where *E*_reactant_, *E*_product_ and *E*_transtion state_ are the gibbs free energy of reactant, product and transtion state respectively.

Tab. S1 Taft spatial steric parameters[1]

| number | Substituent | configuration | Es |
| --- | --- | --- | --- |
| 1 | -H | / | +1.24 |
| 2 | -CH_3_ | / | 0 (standard of reference) |
| 3 | -CH_2_CH_3_ | / | -0.07 |
| 4 | -Cyclohexyl | 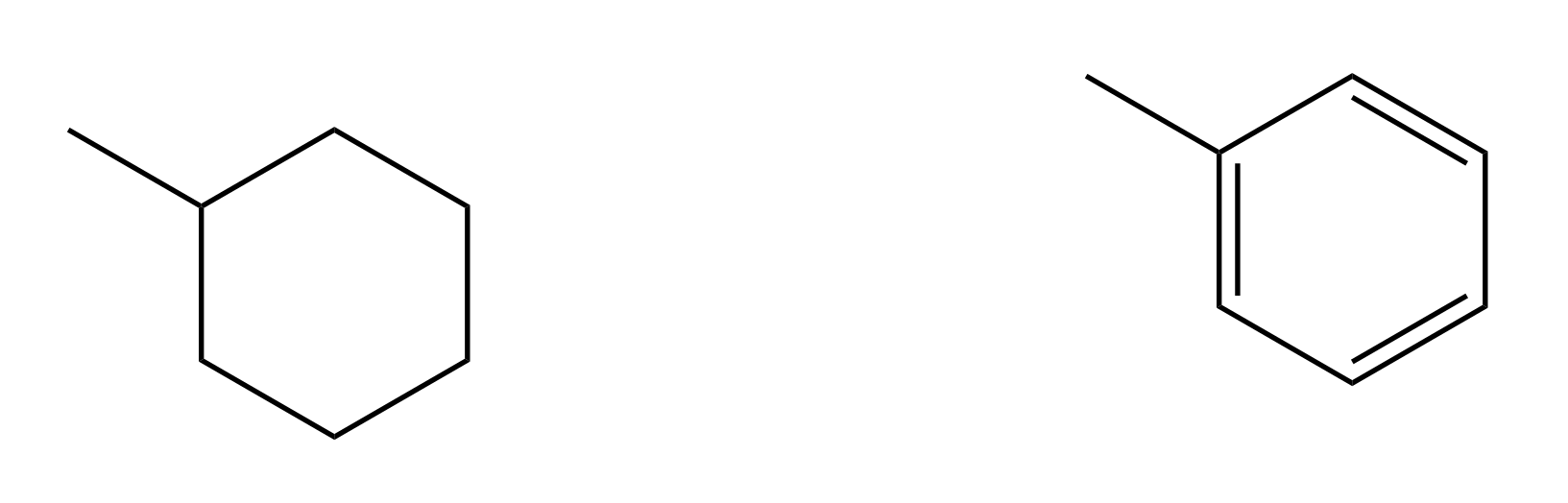 | -0.8 |
| 6 | -Phenyl | 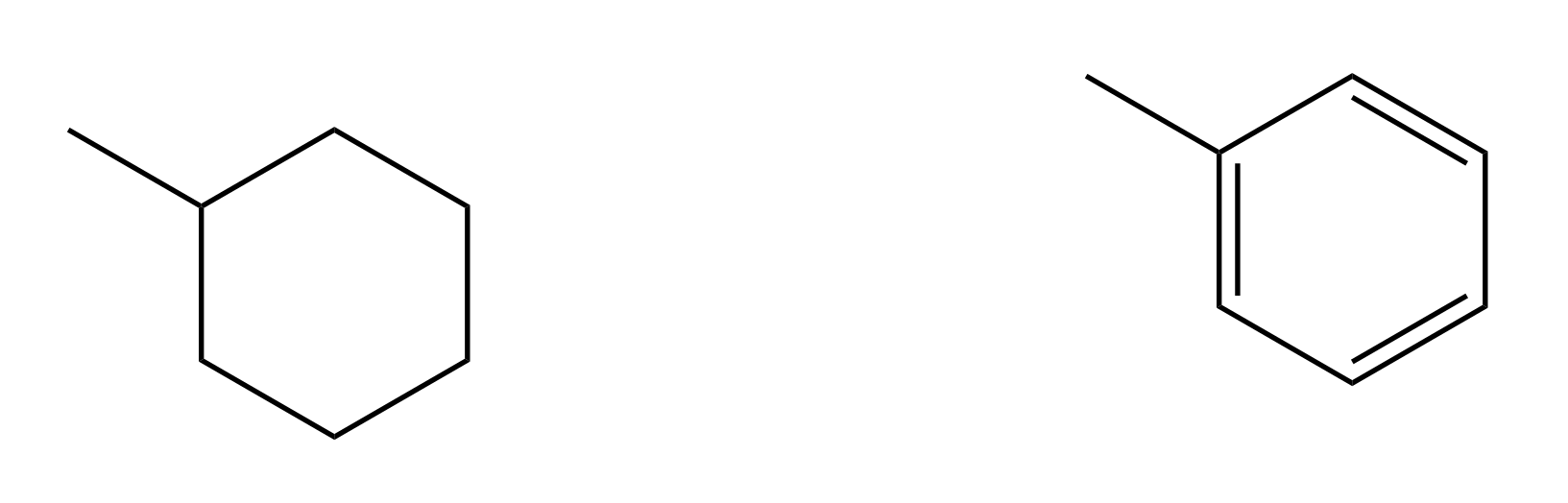 | -0.5 |

[1]RUDOLF ZAHRADNÍK. An Attempt at Calculating the Taft's Constants σ*. Nature 184, 1865 (1959). <https://doi.org/10.1038/1841865a0>

Table S2. Chemicals and gases used in this work and their lowest energy molecular configurations

| Reagent | Abbreviation | CAS | Purity | Boiling point (^o^C) | Optimised molecular configuration |
| --- | --- | --- | --- | --- | --- |
| Carbon dioxide | CO_2_ | 124-38-9 | 99.9% | / | 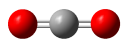 |
| N-Ethylcyclohexylamine | NECHA | 5459-93-8 | ≥99% | 165 | 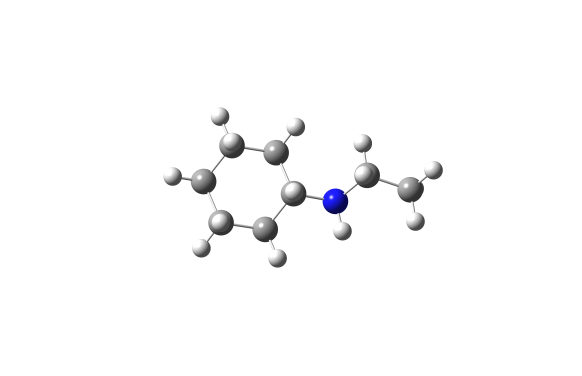 |
| N-Methylcyclohexylamine | NMCHA | 100-60-7 | ≥99% | 149 | 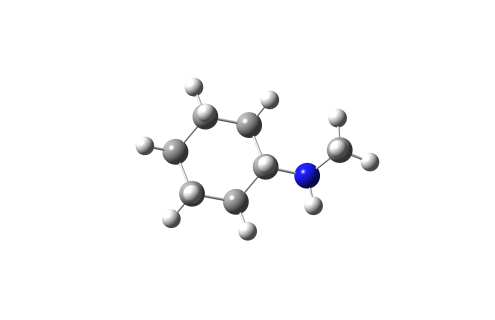 |
| 2,2,6,6-Tetramethylpiperidine | HTMP | 768-66-1 | ≥98% | 152 | 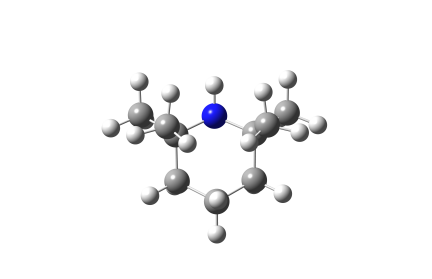 |
| *cis*-2,6-Dimethylpiperidine | CDMP | 766-17-6 | ≥97% | 127 | 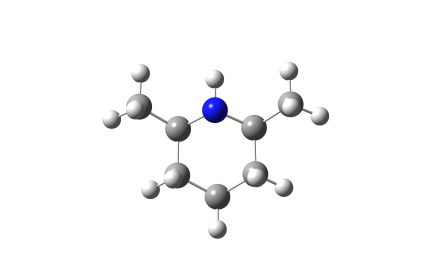 |
| 2-Methylpiperidine | 2-MPD | 109-05-7 | ≥99% | 118 | 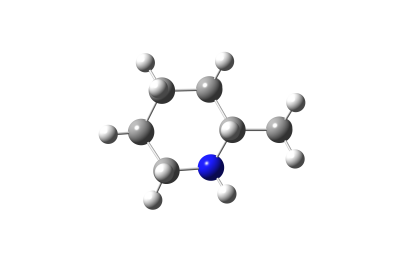 |
| 2-(Isopropylamino)ethanol | IPAE | 109-56-8 | ≥99% | 172 | 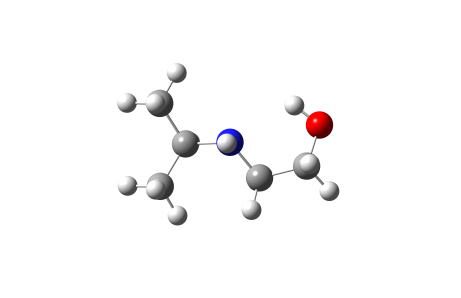 |
| *tert*-Octylamine | TOA | 107-45-9 | ≥98% | 137 | 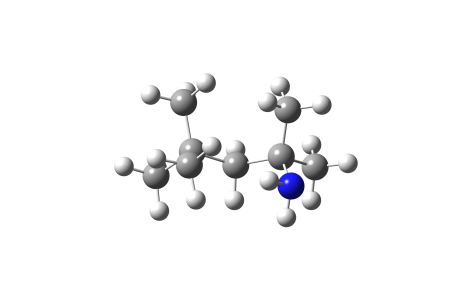 |
| 2-amino-2-methyl-1-propanol | AMP | 124-68-5 | ≥95.0% | 165 | 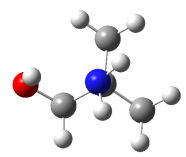 |
| Monoethanolamine | MEA | 141-43-5 | ≥99% | 170 | 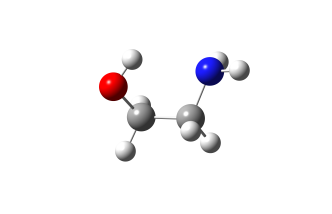 |
| Cyclohexylamine | CHA | 108-91-8 | ≥99% | 134 | 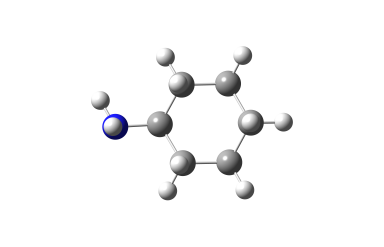 |
| 3-methylmorpholine | 3-MMP | 42185-06-8 | ≥97% | 133 | 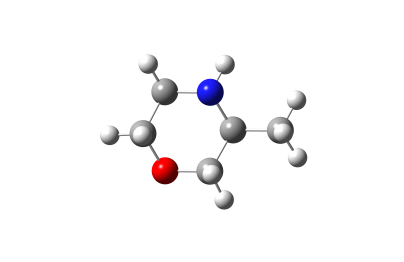 |
| Morpholine | Morp | 110-91-8 | ≥99.5% | 126 | 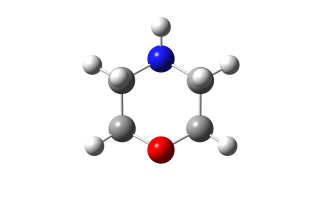 |
| N-Methylaniline | NMAB | 100-61-8 | ≥98% | 196 | 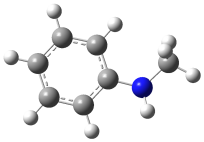 |
| Aminobenzene | AB | 62-53-3 | ≥99% | 184 | 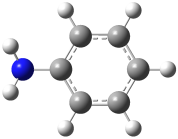 |
| Ethylene Glycol | EG | 107-21-1 | >99.0% | 195 | 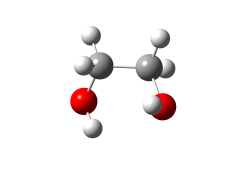 |
| Dimethylsulfoxide-d6 | DMSO-d6 | 2206-27-1 | 99.9% (TMS 0.03%) | / | / |
| Methanol-d4 | CD_3_OD | 811-98-3 | 99.8% (TMS 0.03%) | / | / |
| Hydrochloric acid standard solution | / | / | 1 mol L^–1^ | / | / |

Tab. S3 Sterically hindered amines screened out due to low boiling points (≤100 ^o^C)

| number | Sterically hindered amines | CAS | Boiling point (^o^C) | Molecular structure |
| --- | --- | --- | --- | --- |
| 1 | tert-Butylamine | 75-64-9 | 46 | 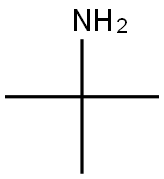 |
| 2 | tert-Amylamine | 594-39-8 | 77 | 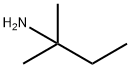 |
| 3 | 3-Amino-3-methyl-1-butanol | 42514-50-1 | 71 | 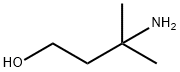 |
| 4 | N-Methyl-2-methyl-2-propanamine | 14610-37-8 | 66 | 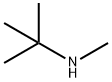 |
| 5 | N-tert-Butylethylamine | 4432-77-3 | 89 | 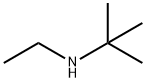 |
| 6 | N-tert-Butylisopropylamine | 7515-80-2 | 98 | 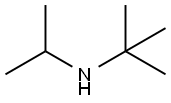 |
| 7 | Di-tert-butylamine | 21981-37-3 | 92 | 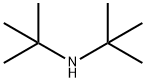 |
| 8 | 2-(tert-Butylamino)ethanol | 4620-70-6 | 90 | 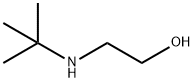 |
| 9 | 1-[(1,1-Dimethylethyl)amino]-2-propanol | 18366-38-6 | 58 | 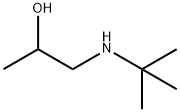 |
| 10 | N-Ethyl-2-propanamin | 19961-27-4 | 71 | 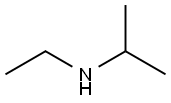 |
| 11 | Diisopropylamine | 108-18-9 | 84 | 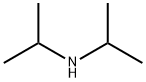 |
| 12 | N-Isopropylpropan-1-amine | 21968-17-2 | 92 | 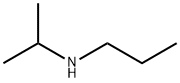 |
| 13 | N-Isopropylalaninol | 24403-02-9 | 71 | 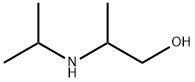 |
| 14 | 2-Amino-2-methylpentanenitrile | 58577-08-5 | 87 | 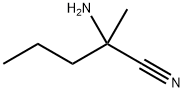 |
| 15 | N-Isopropylcyclohexylamine | 1195-42-2 | 60 | 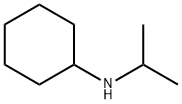 |
| 16 | N-Isopropylaniline | 212-196-7 | 55 | 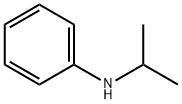 |
| 17 | 2-Propylpiperidine | 3238-60-6 | 50 | 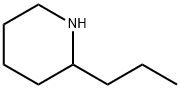 |
| 18 | 2-(Hydroxymethyl)piperidine | 3433-37-2 | 100 | 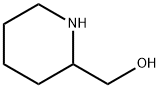 |

Tab. S4 Sterically hindered amines screened out due to high prices or lack of access to purchases

| number | Sterically hindered amines | CAS | boiling point (^o^C) | Price (¥) | Molecular structure |
| --- | --- | --- | --- | --- | --- |
| 1 | 2-Methylpentan-2-amine | 53310-02-4 | 102 | no | 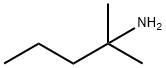 |
| 2 | 3-Amino-3-methyl-2-butanol | 13325-14-9 | 160 | 1522/g | 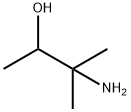 |
| 3 | 2-Amino-2-methyl-3-pentanol | 89585-20-6 | 199 | no | 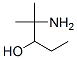 |
| 4 | 2-Methyl-N-propylpropan-2-amine | 22675-81-6 | 112 | no | 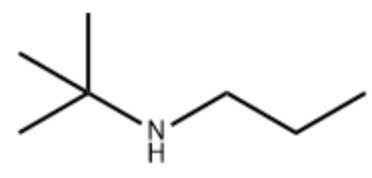 |
| 5 | N-sec-Butylpropylamine | 39190-67-5 | 123 | 70/mL | 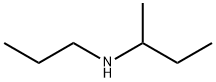 |
| 6 | 2-Methyl-3-methoxypropan-2-amine | 20719-68-0 | 101 | 850/g | 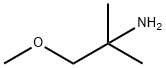 |
| 7 | 1-Methylcyclohexanamine | 6526-78-9 | 143 | 2390/g | 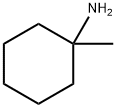 |
| 8 | N-Cyclohexyl-N-propylamine | 3592-81-2 | 191 | 1538/g | 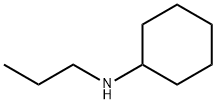 |
| 9 | N-(sec-Butyl)cyclohexanamine | 42966-62-1 | 193 | no | 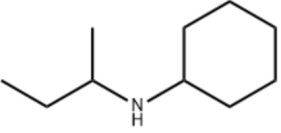 |
| 10 | N-tert-Butylcyclohexylamine | 51609-06-4 | 172 | 1465/mL | 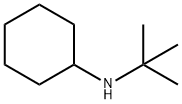 |
| 11 | 2-Ethylpiperidine | 1484-80-6 | 143 | 90/mL | 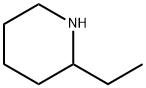 |
| 12 | 2-(1-Methylethyl)piperidine | 22977-56-6 | 162 | no | 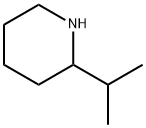 |
| 13 | 2,2-Dimethylpiperidine | 54152-47-5 | 135 | no | 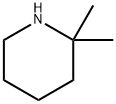 |
| 14 | 2-Piperidinol | 45506-41-0 | 180 | 57/g | 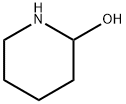 |
| 15 | Rac-[(2r,6r)-6-methylpiperidin-2-yl]methanol | 38299-74-0 | 206 | no | 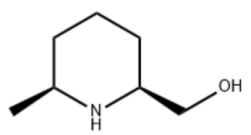 |
| 16 | 3,3-Dimethylmorpholine | 59229-63-9 | 143 | 185/g | 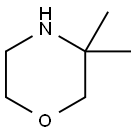 |
| 17 | 3-Ethylmorpholine hydrochloride | 55265-24-2 | 157 | 1222/g | 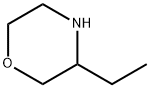 |
| 18 | 3-Morpholinylmethanol | 106910-83-2 | 173 | 364/g | 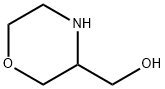 |
| 19 | (S,S)-3,5-Dimethylmorpholine | 154634-96-5 | 151 | 579/g | 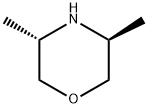 |

Tab. S5 Sterically hindered amines screened out due to low solubility in EG

| number | Sterically hindered amines | CAS | Boiling point (^o^C) | Molecular structure |
| --- | --- | --- | --- | --- |
| 1 | 2-Amino-2-methyl-1,3-propanediol | 115-69-5 | 151 | 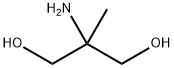 |
| 2 | 2-Amino-2-ethyl-1,3-propanediol | 115-70-8 | 152 | 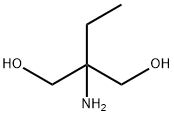 |
| 3 | Tris(hydroxymethyl)aminomethane | 77-86-1 | 219 | 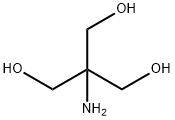 |
| 4 | Dicyclohexylamine | 101-83-7 | 256 | 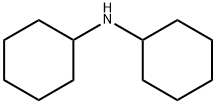 |
| 5 | N,N-Bis(4-tert-butylphenyl)amine | 4627-22-9 | 195 | 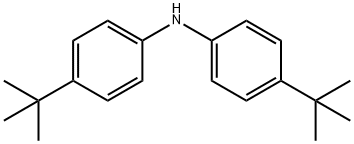 |
| 6 | Piperazine organic amines are all excluded due to their lower solubility and are not developed here. | | | |


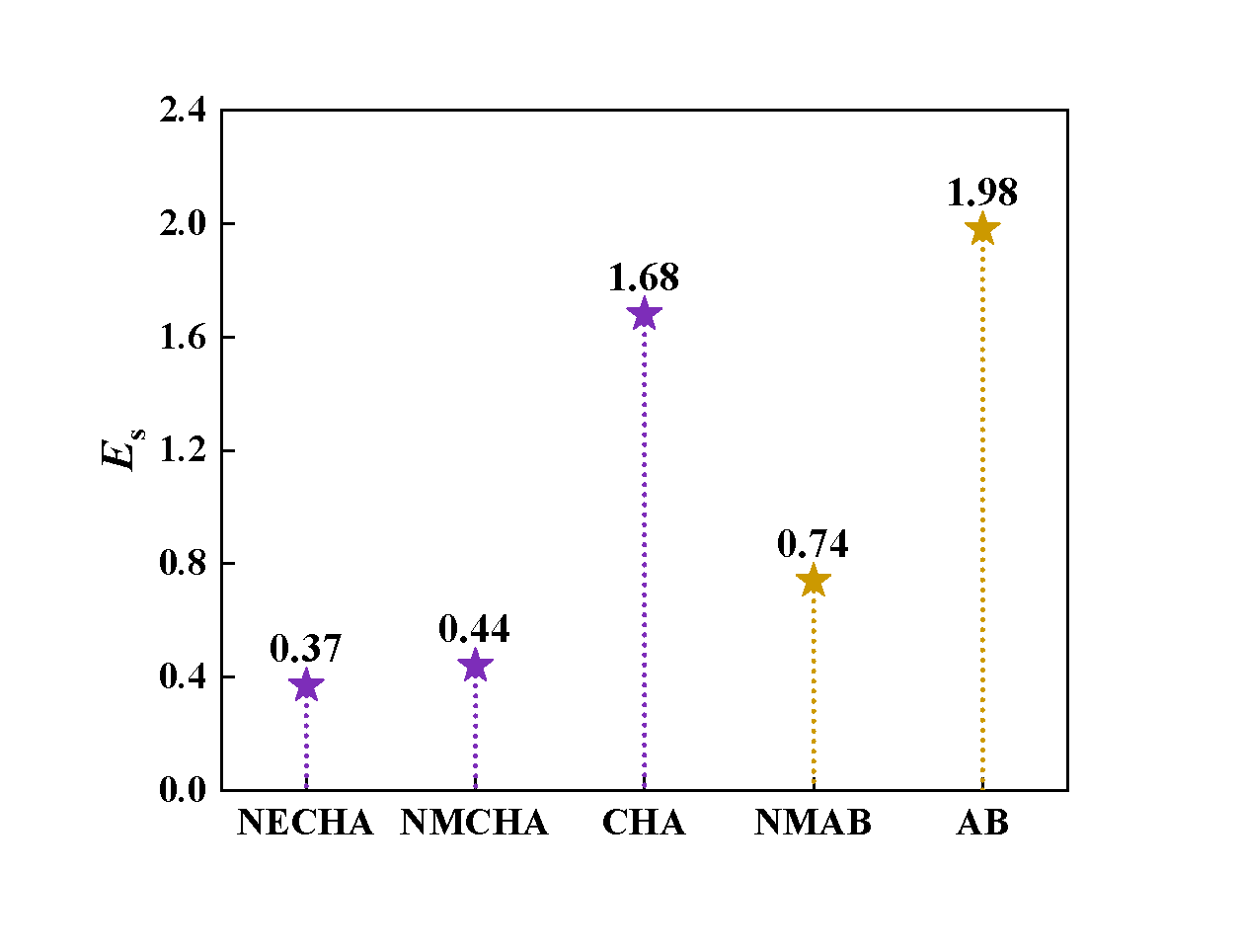


Fig. S1 *E*_s_ values of organic amines NECHA, NMCHA, CHA, NMAB, and AB


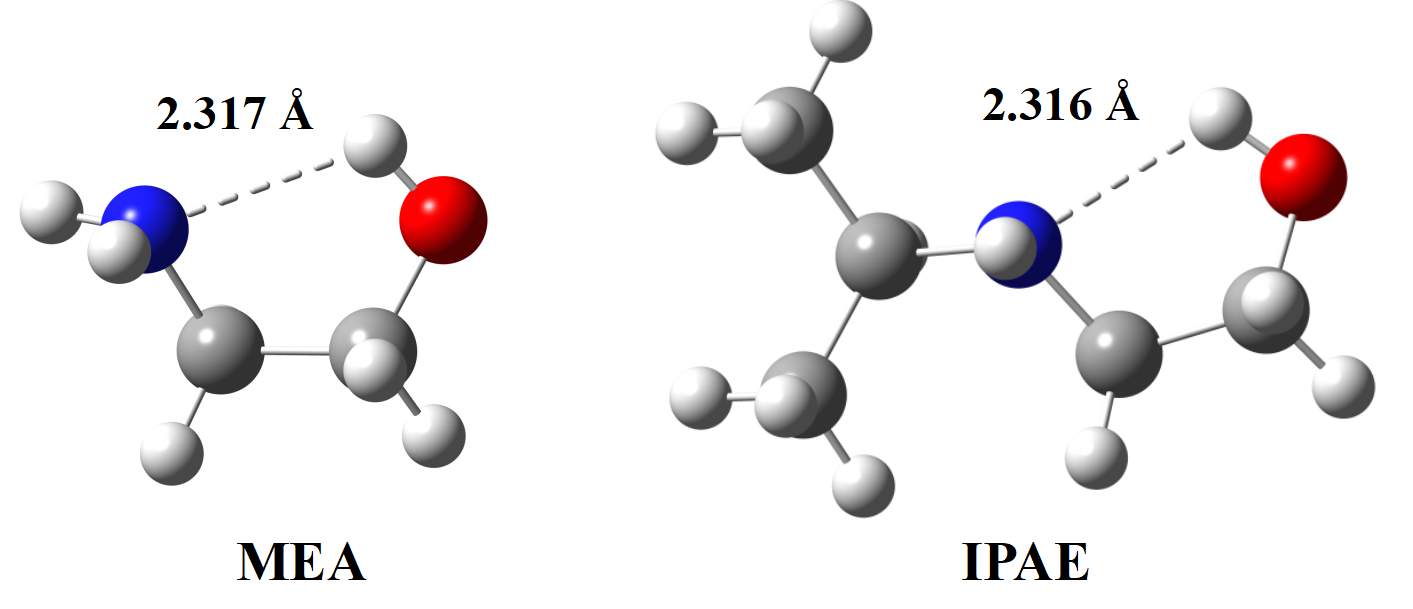


Fig. S2 Intramolecular hydrogen bonds of MEA and IPAE


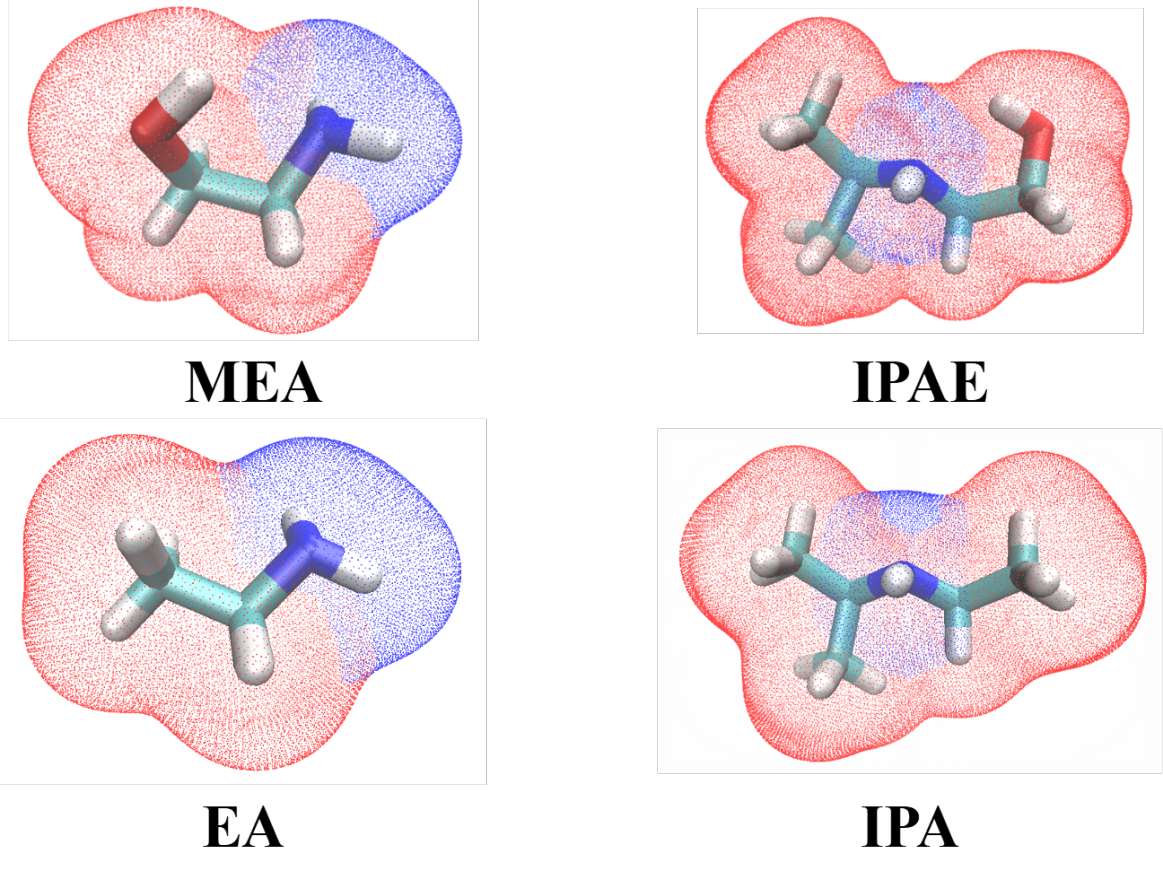


Fig. S3 The comparison of vdW SA between MEA, IPAE and dehydroxylated derivatives EA, IPA


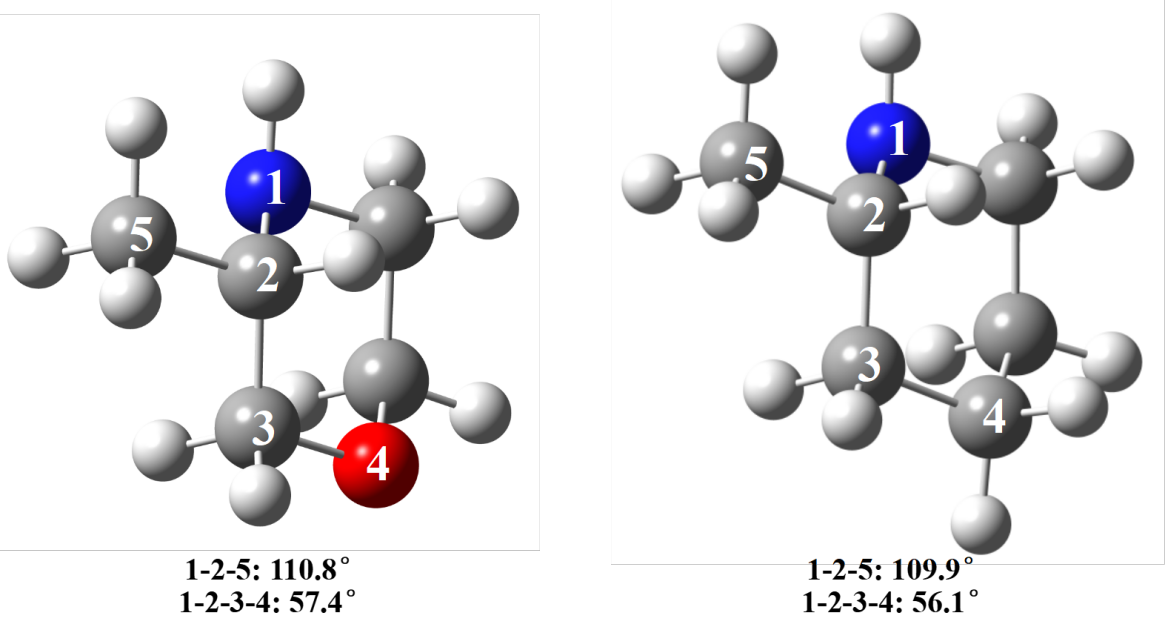


Fig. S4 Structural comparison between 3-MMP (left) and 2-MPD (right)

Fig. S5 Relative molecular mass of organic amines


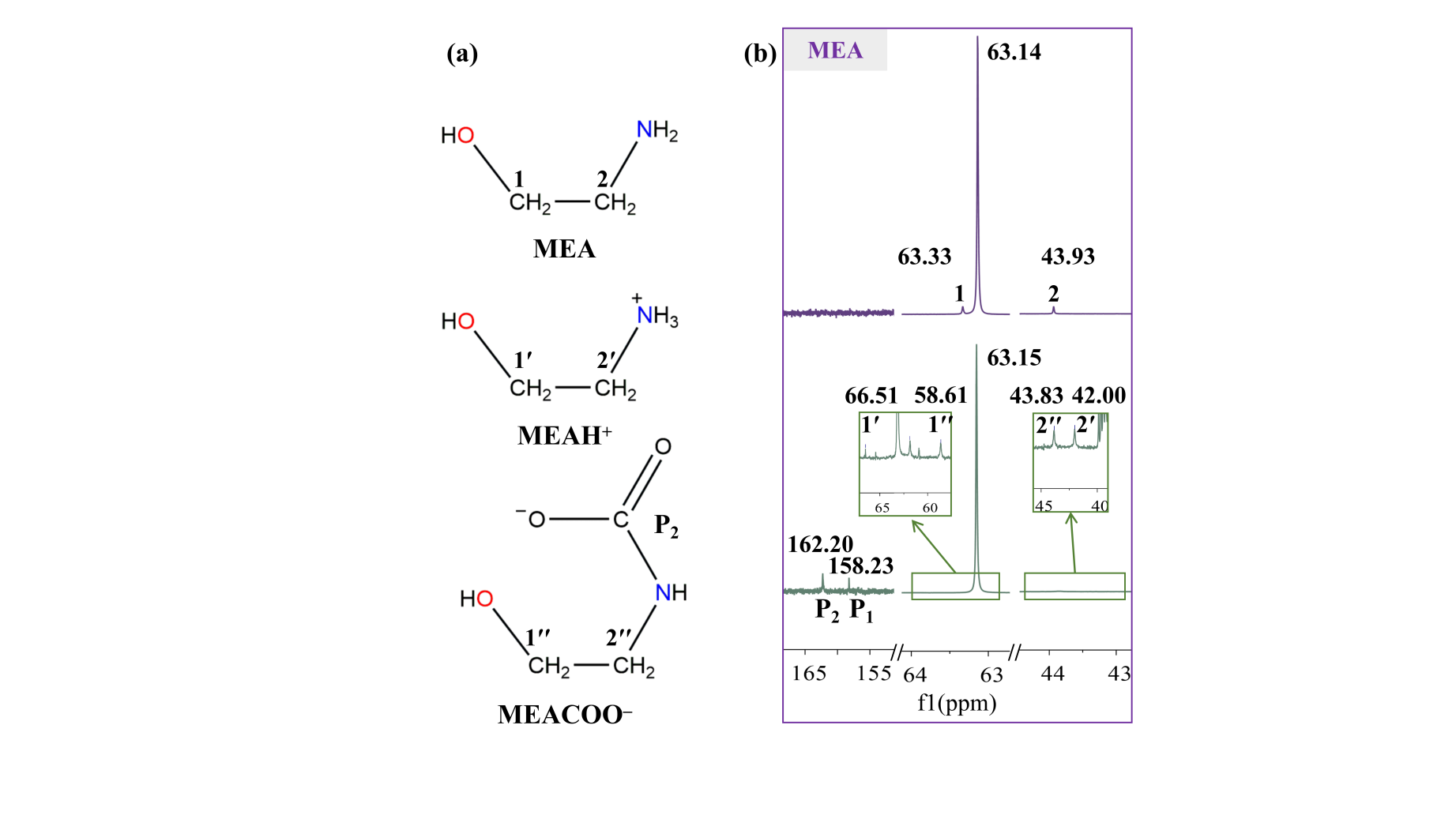


Fig. S6 ^13^C NMR spectra of MEA before (upper) and after (lower) reaction


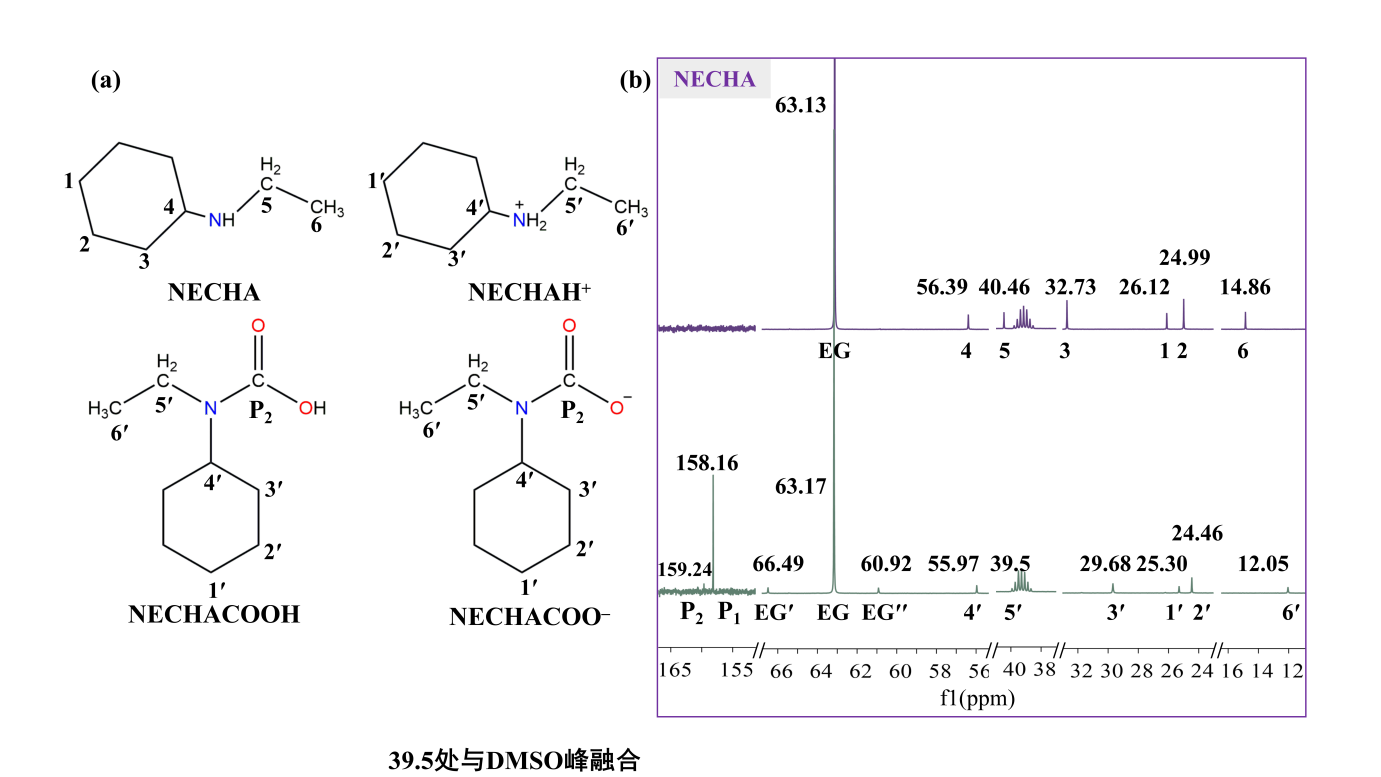


Fig. S7 ^13^C NECHA spectra of NECHA before (upper) and after (lower) reaction


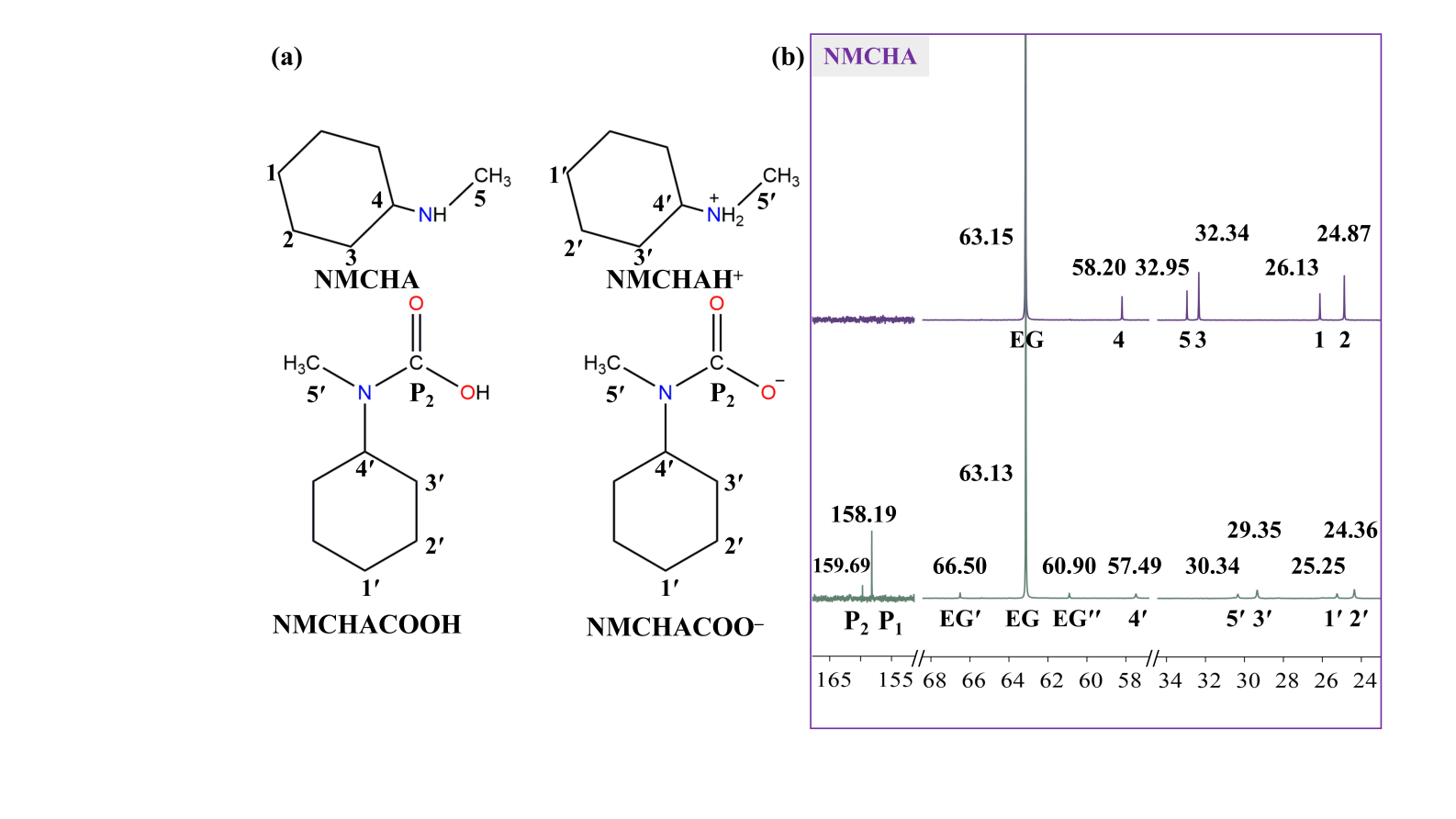


Fig. S8 ^13^C NMR spectra of NMCHA before (upper) and after (lower) reaction


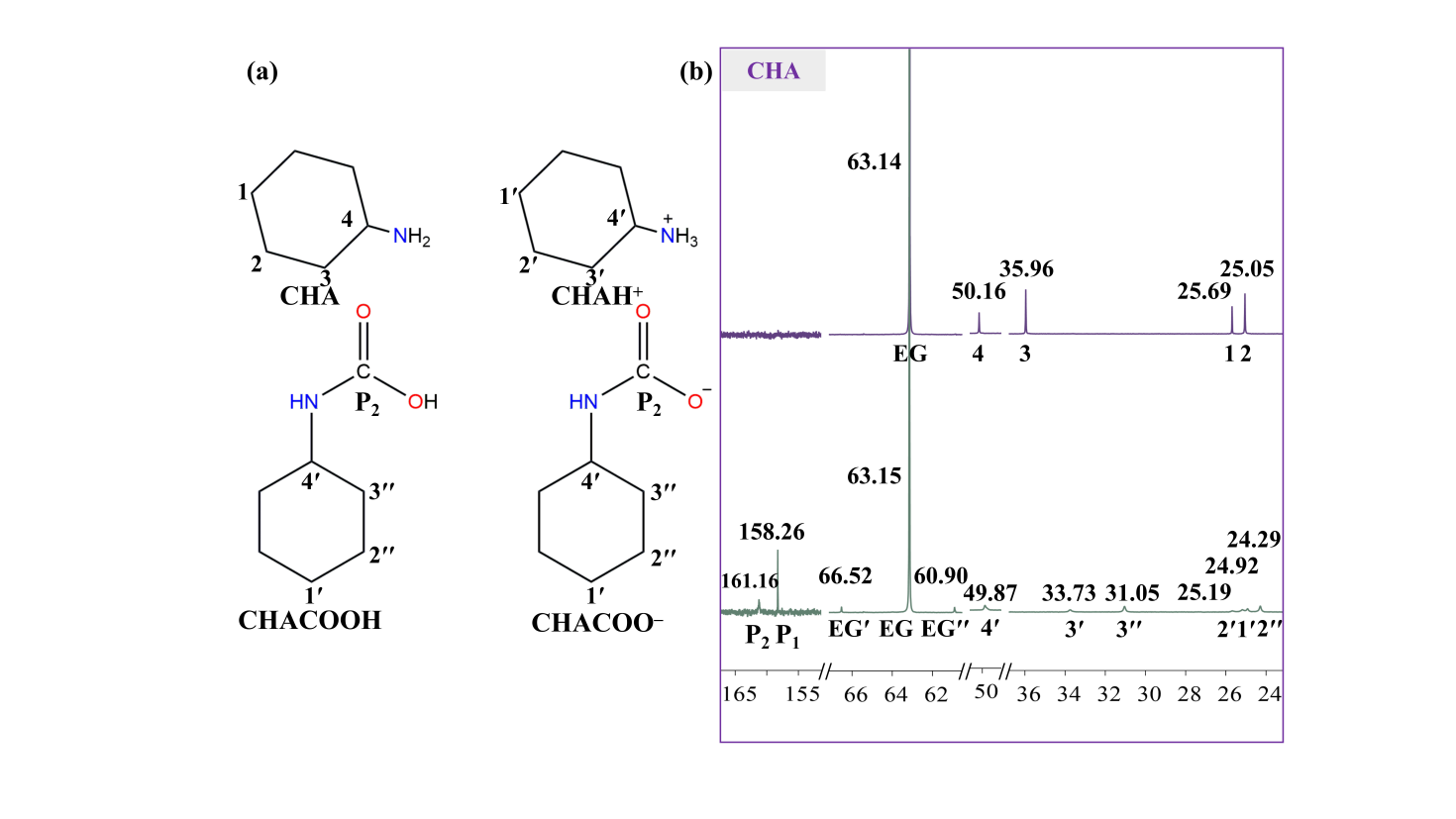


Fig. S9 ^13^C NMR spectra of CHA before (upper) and after (lower) reaction


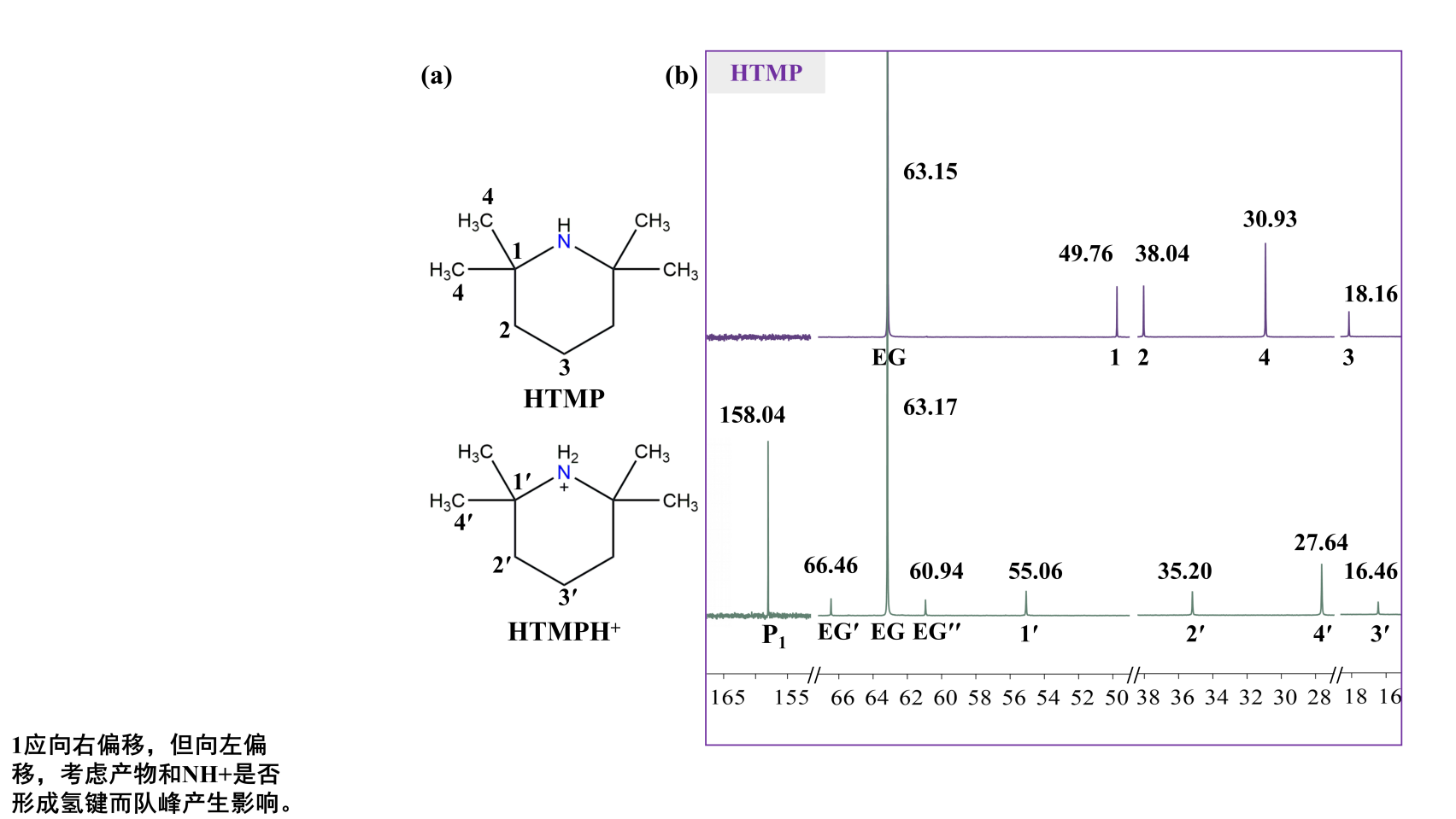


Fig. S10 ^13^C NMR spectra of HTMP before (upper) and after (lower) reaction


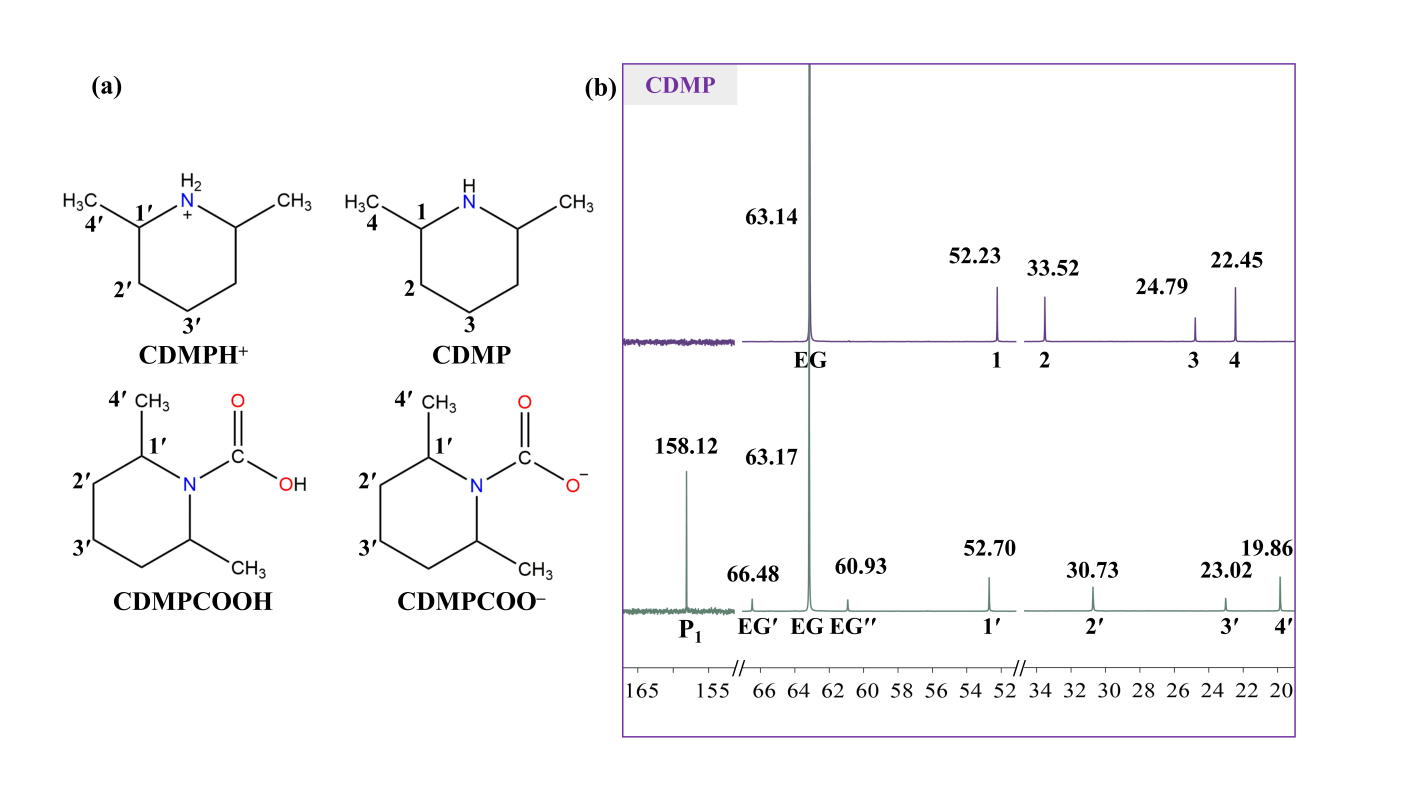


Fig. S11 ^13^C NMR spectra of CDMP before (upper) and after (lower) reaction


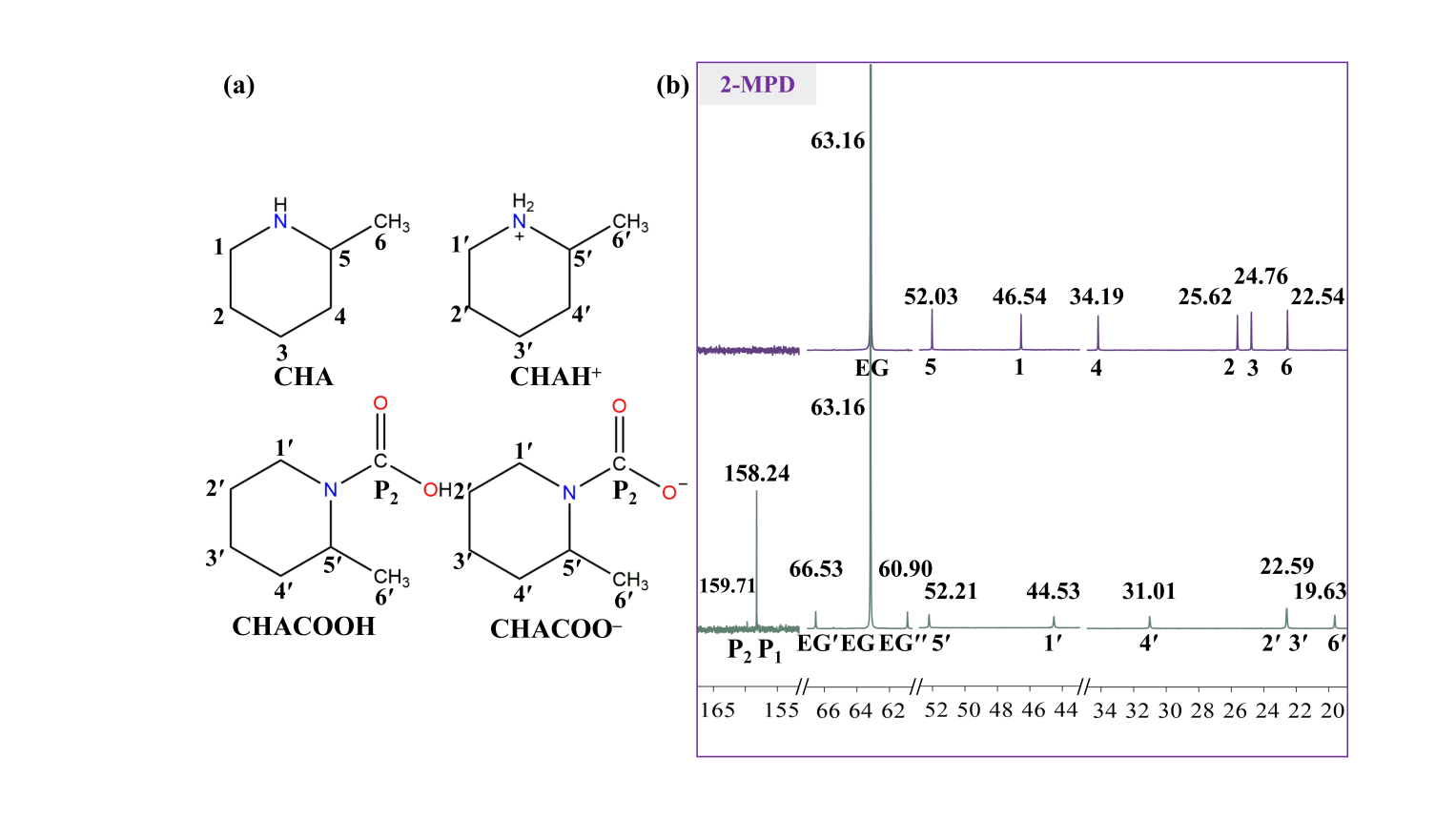


Fig. S12 ^13^C NMR spectra of 2-MPD before (upper) and after (lower) reaction


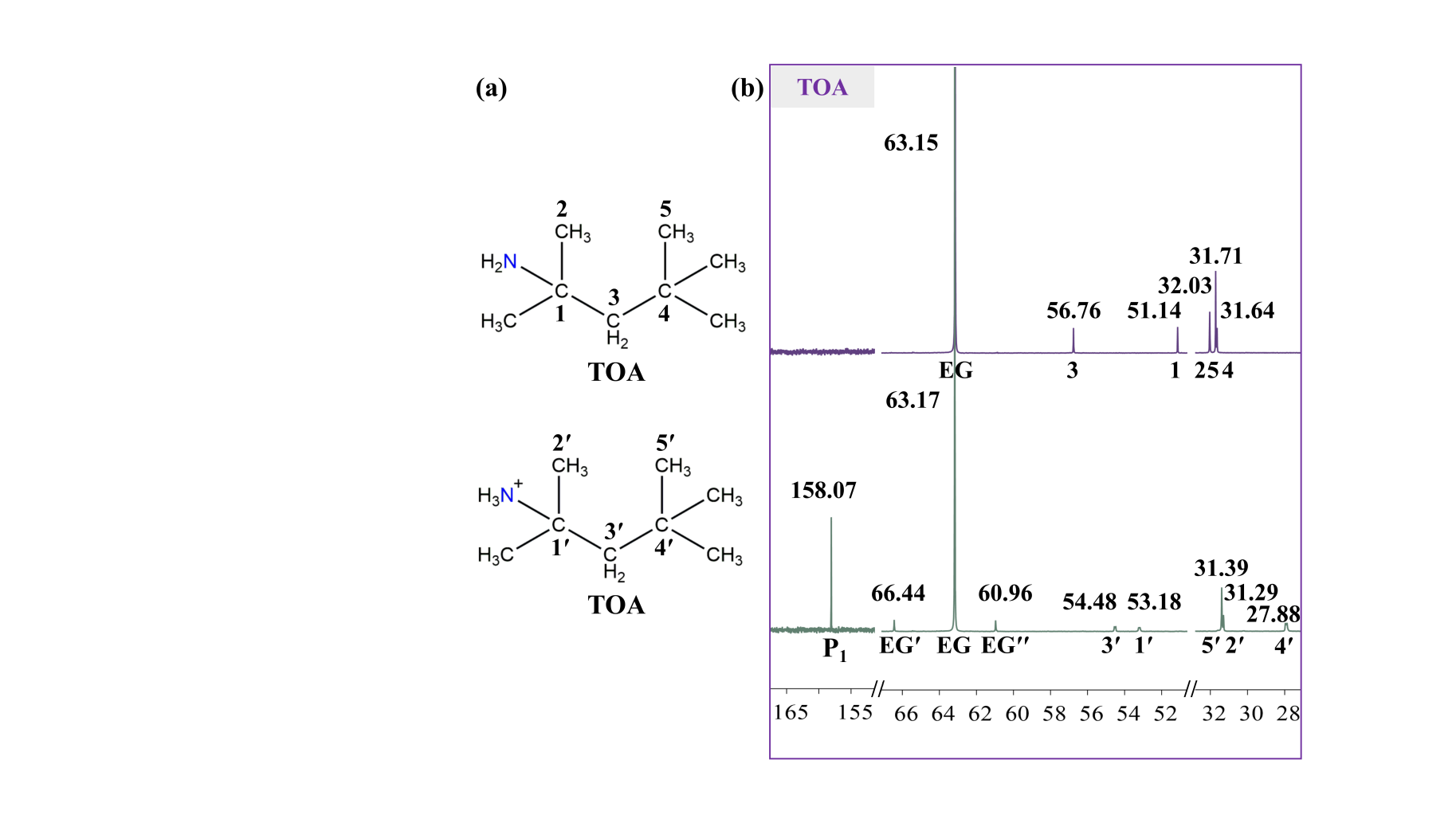


Fig. S13 ^13^C NMR spectra of TOA before (upper) and after (lower) reaction


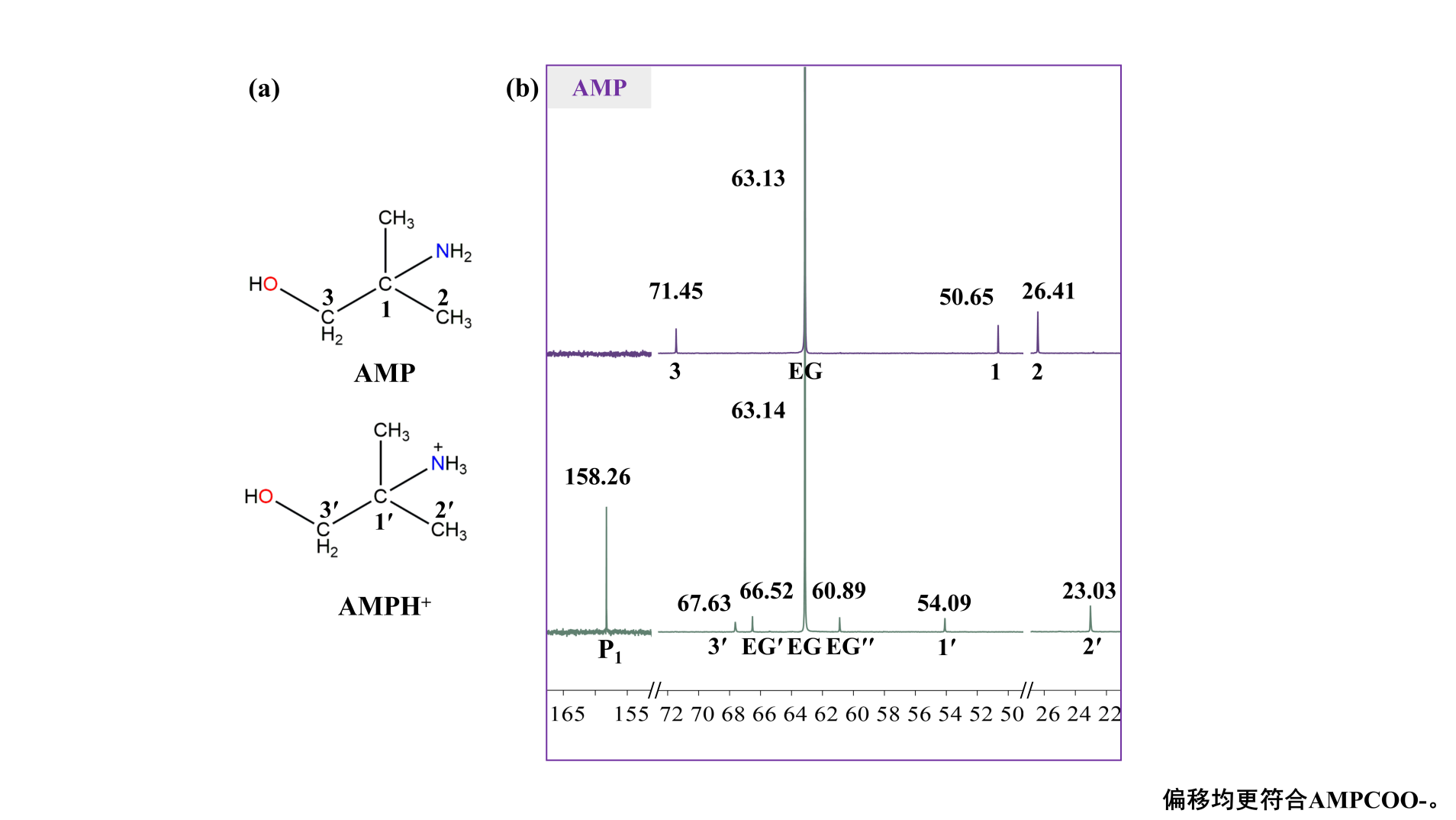


Fig. S14 ^13^C NMR spectra of AMP before (upper) and after (lower) reaction


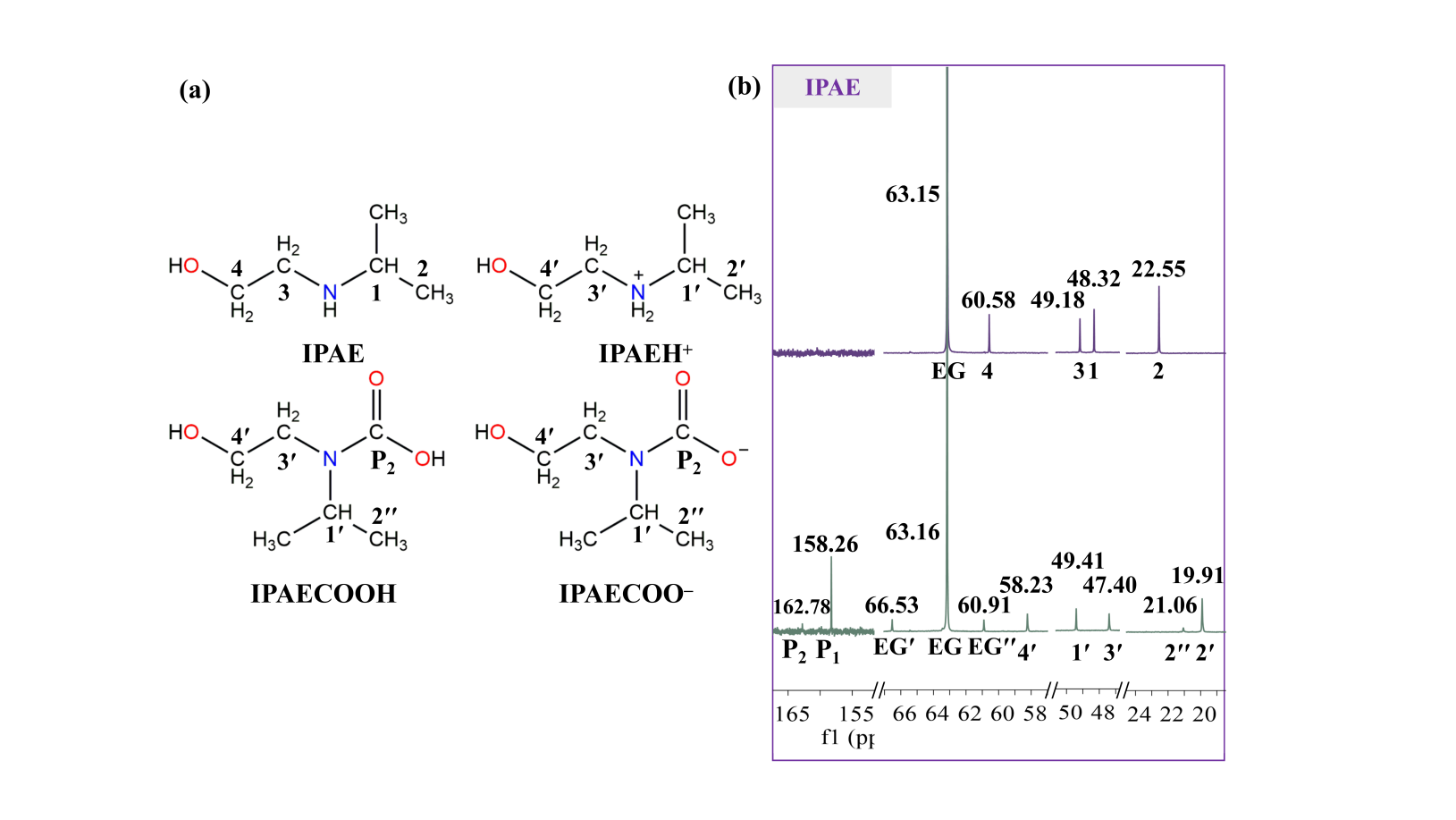


Fig. S15 ^13^C NMR spectra of IPAE before (upper) and after (lower) reaction


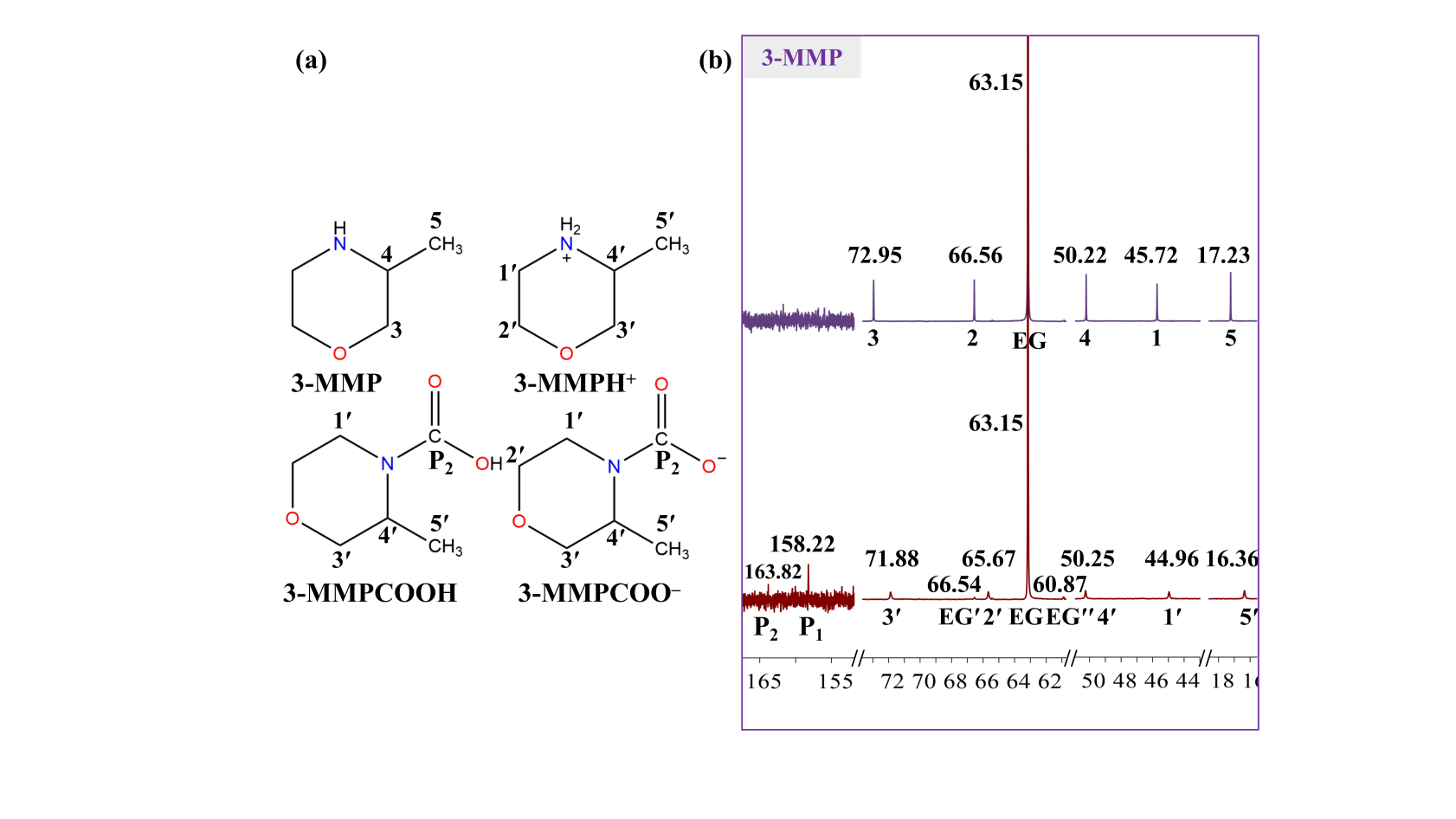


Fig. S16 ^13^C NMR spectra of 3-MMP before (upper) and after (lower) reaction


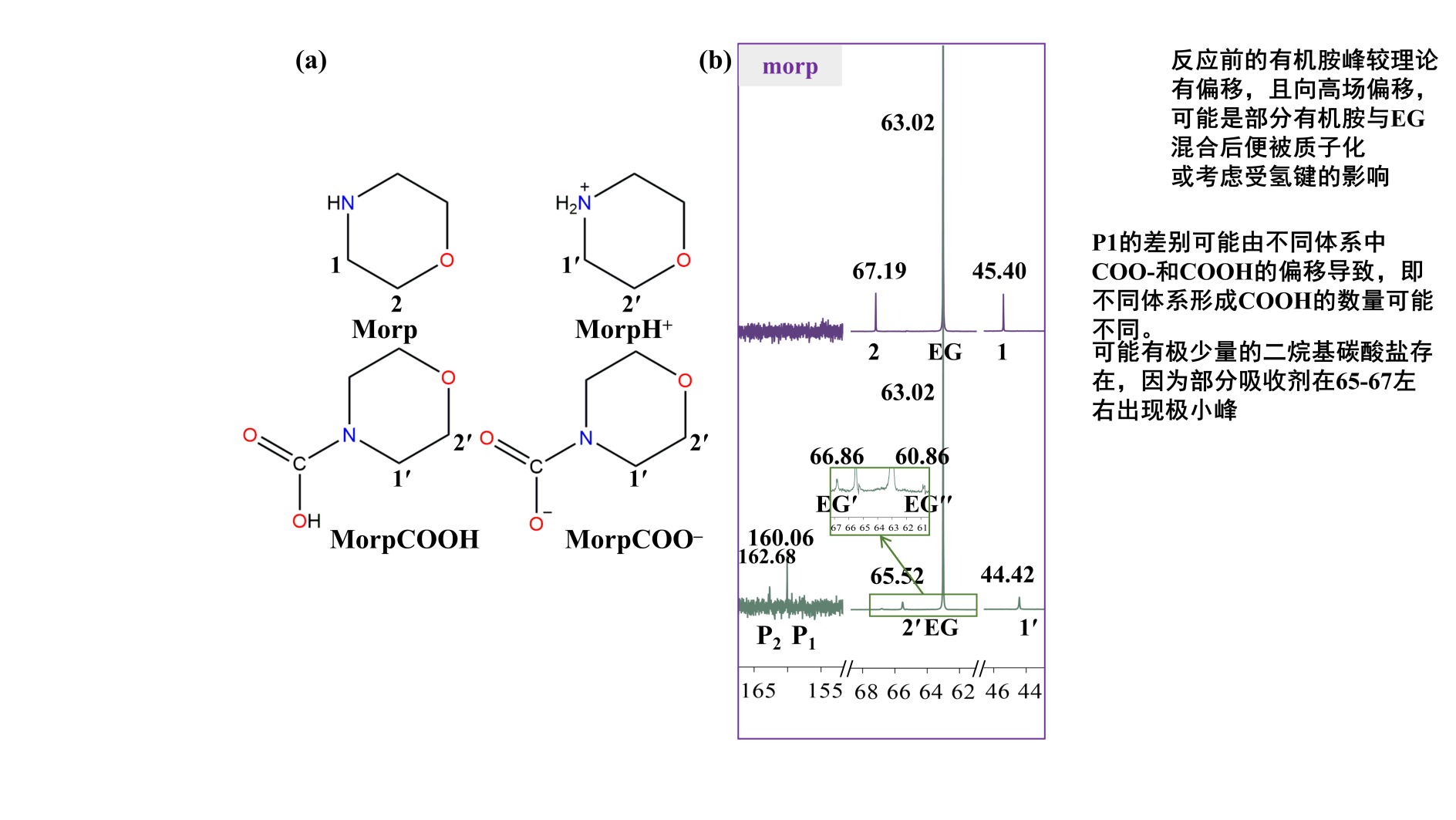


Fig. S17 ^13^C NMR spectra of Morp before (upper) and after (lower) reaction


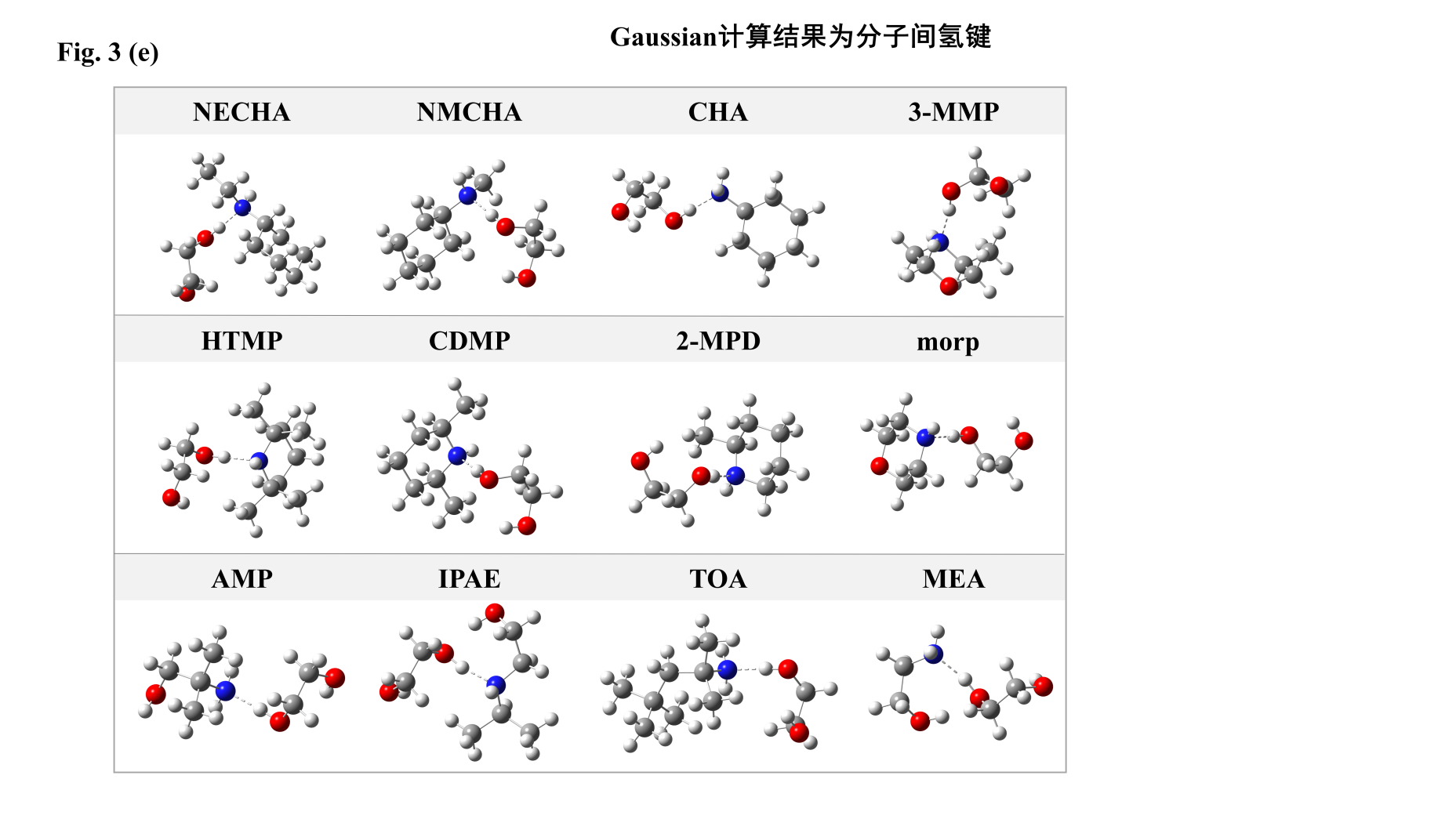


Fig. S18 Hydrogen bonds formed between organic amines and EG

Fig. S19 The energy barriers for Step 1 and Step 2

Fig. S20 The Gibbs free energy change for Step 1, Step 2, and overall reaction

Fig. S21 Maximum heat flow values for absorbents

Fig. S22 Total heat release for absorbents


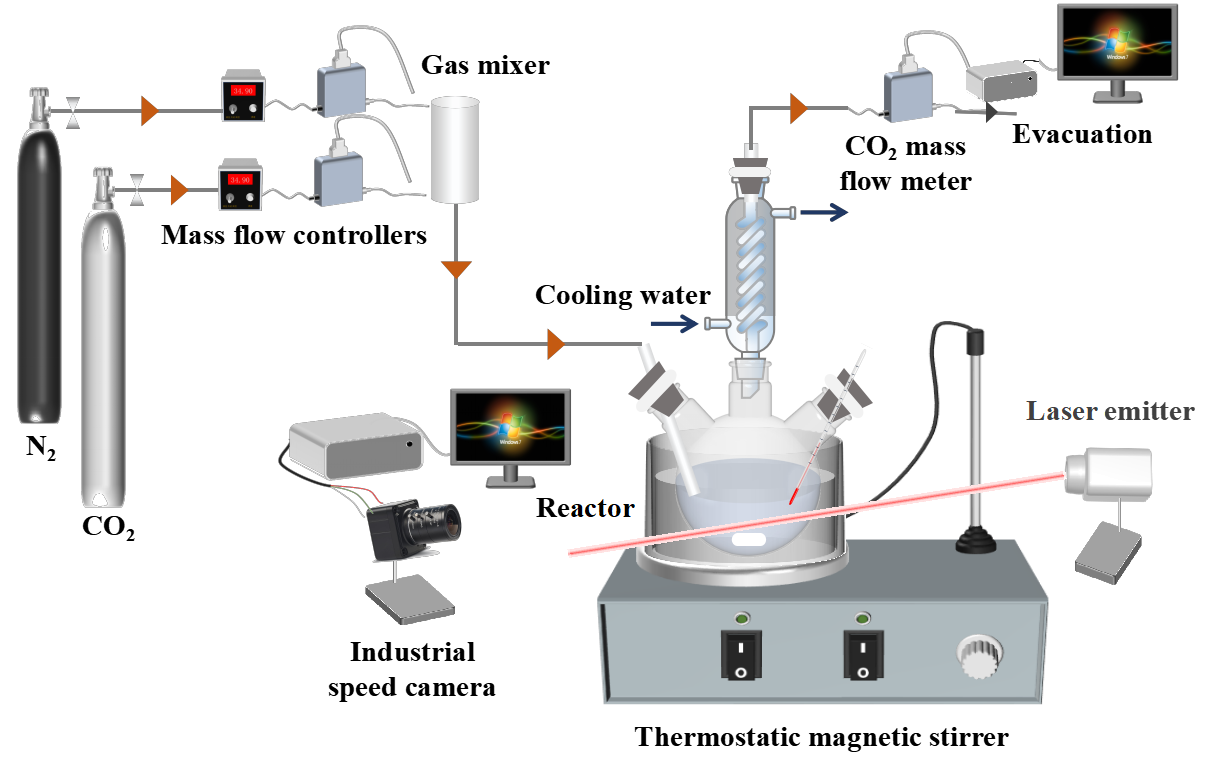


Fig. S23 Schematic diagram of CO_2_ absorption and desorption apparatus


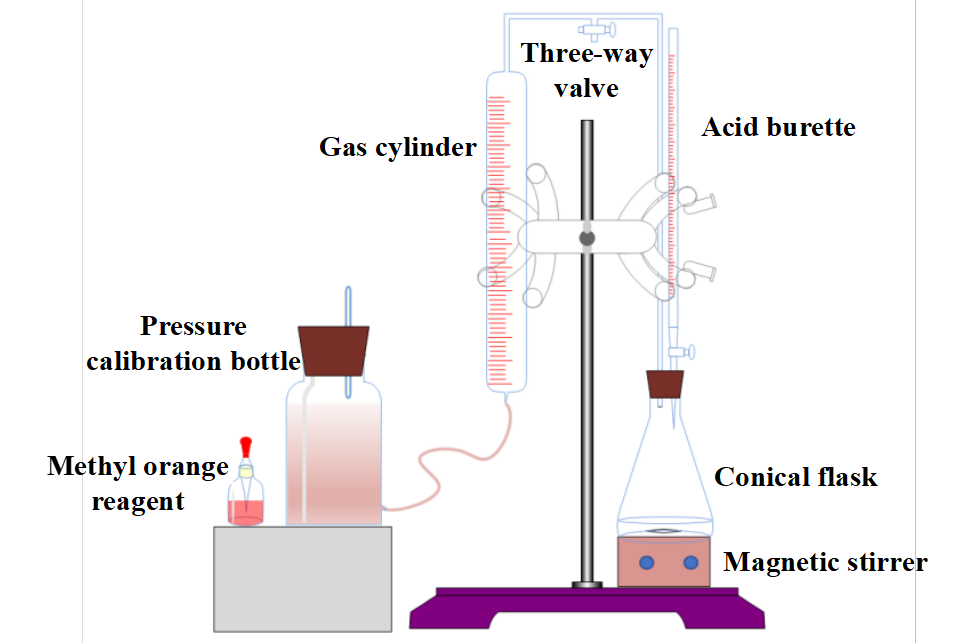


Fig. S24 Chittick apparatus
